# Advancements in Inkjet Printing of Metal- and Covalent-Organic Frameworks: Process Design and Ink Optimization

**DOI:** 10.1021/acsami.4c15957

**Published:** 2025-02-14

**Authors:** Seyyed Abbas Noorian Najafabadi, Chunyu Huang, Kaï Betlem, Thijmen A. van Voorthuizen, Louis C. P. M. de Smet, Murali Krishna Ghatkesar, Martijn van Dongen, Monique Ann van der Veen

**Affiliations:** † Chemical Engineering Department, Delft University of Technology, 2629 HZ Delft, The Netherlands; ‡ Department of Chemical Sciences, University of Padova, 35131 Padova, Italy; ¶ Department of Microelectronics, Delft University of Technology, 2628 CD Delft,The Netherlands; § Department of Precision and Microsystems Engineering, Delft University of Technology, 2628 CD Delft, The Netherlands; ∥ Laboratory of Organic Chemistry, Wageningen University and Research, 6708 WE Wageningen, The Netherlands; ⊥ Research Group Applied Natural Sciences, Fontys University of Applied Sciences, 5600 AH Eindhoven, The Netherlands

**Keywords:** Metal-organic frameworks, Covalent-organic frameworks, Inkjet printing, Patterns, Films

## Abstract

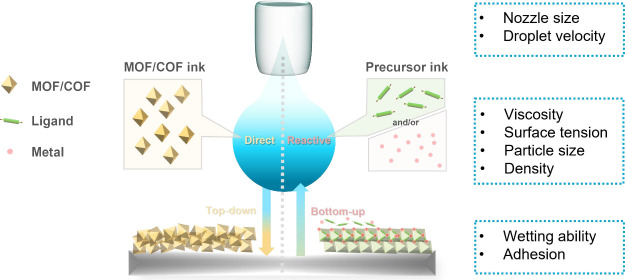

Metal-organic frameworks (MOFs) and covalent-organic frameworks (COFs) are highly versatile materials based on inorganic modes connected via organic linkers or purely via the connection of organic building blocks, respectively. This results in 3-D nanoporous frameworks, which, due to their combination of high porosity and variability of building blocks, can exhibit exceptional properties that make them attractive. Certain applications (e.g., in electronics and as membranes) require a thin film or even a patterned morphology on various substrates. Inkjet printing of MOFs has emerged as a simple and effective technique for the scalable production of a wide range of MOF (gradient) films and patterns on a wide range of substrates according to specific requirements. This review comprehensively reviews the achievements in inkjet printing of both MOFs and COFs. We discuss the different substrates, ink formulation, and hardware intertwined requirements needed to achieve high-resolution printing and obtain desired properties such as porosity, physical-mechanical characteristics, and uniform thickness. Crucial aspects related to ink formulation, such as colloidal stability and size control of MOFs and COFs, are discussed. Additionally, we highlight potential opportunities for furthering the development of inkjet printing of MOFs/COFs and critically assess the reporting of the printing procedures and characterization of the resultant materials. In this manner, this review aims to contribute to the advancements in understanding and optimization of inkjet printing of MOFs and COFs, as this technique holds great potential for diverse applications and functionalization of MOF/COF films and patterns.

## 1 Introduction

Metal–organic frameworks (MOFs) are a versatile class of porous materials consisting of metal ions or clusters coordinated with organic ligands, creating highly tunable structures with features such as low density, high surface area, regular porosity, and chemical adaptability.^1−3^ The adaptability of MOFs stems from the careful selection of metal ions/clusters and organic ligands, which allows for tailored structures with numerous active sites. This versatility makes MOFs ideal for various applications, including adsorption, catalysis, sensing, and drug delivery.^4,5^ Similarly, covalent organic frameworks (COFs) that are constructed via organic building blocks (such as boronate ester and imine bonds, rather than metal-organic coordination) share structural and functional parallels with MOFs. Particularly, COFs also show high surface area and regular porosity, which also makes them suitable for applications such as catalysis, gas storage, and drug delivery. However, unlike MOFs, greater challenges are posed to achieve high crystallinity for COFs.^6^

Though MOFs and COFs are most often characterized and used in powder form, certain applications such as optics,^7^ photonics,^8^ electronics,^9,10^ optoelectronics,^10−12^ sensing,^13,14^ catalytic coatings,^15^ membranes,^16−19^ solar cells,^20^ batteries,^21^ and supercapacitors^22^ require a thin layer or pattern on the surface. Ideally, MOFs and COFs will still impart their extended surface area in film form, thus enabling higher adsorption capacity, enhanced catalytic activity, and improved sensitivity in these applications compared to dense materials.^23−25^

Various methods are employed to fabricate MOF or COF films on substrates, such as drop-casting,^26,27^ layer-by-layer assembly,^28,29^ chemical vapor deposition,^30,31^ roll-to-roll printing,^32^ dip-coating,^33,34^ spray-coating,^35,36^ and spin-coating.^37,38^ These methods have their advantages and disadvantages, depending on the solution and substrates being used. For large-scale coatings, roll-coating is widely used in industry for its efficiency in material utilization. Spray-coating techniques, with no substrate size restrictions and minimal polymer use, present a promising alternative for large-scale manufacturing. Despite this potential, spray coating often results in thicker and more uneven layers, limiting its application. Dip-coating offers a quick, easy, affordable, and high-quality coating method for both industrial and lab applications. However, there is a significant loss of the precursor solution. Layer-by-layer (LbL) assembly and chemical vapor deposition (CVD) techniques involve complex, multistep procedures that often require expensive and sophisticated machinery, take a long time, produce significant amounts of chemical waste, and are typically limited to relatively small areas.^39,40^

Additionally, methods such as spray coating^41,42^ and deposition using microfluidic devices^43−45^ allow for precise layer-by-layer control over film deposition, further enhancing control over film composition and thickness. Inkjet printing, in contrast, is more material efficient and offers more adaptability (e.g., gradients in films can be easily achieved^39^ and is more convenient to use compared to LbL- and CVD-based techniques). Inkjet printing enables deposition of various MOF or COF sensing materials in picoliter droplets.^46,47^ The framework material is added to a suitable solvent(s), and the solution is adjusted to the desired viscosity and surface tension to dispense as ink droplets. After droplet deposition on the surface, the solvent evaporates and a layer of MOF or COF material remains. The spread of the material depends on how the droplet wets the surface as well as the rate of droplet evaporation, which in turn depends on the surface energy and droplet volume, respectively.^48^ Inkjet printing gives precise volume control, reproducibility, and high printing throughput.^49^ Droplets can be deposited on a desired surface at a chosen location, giving control on functionalizing micro/nanoscale sensing surfaces.^50^ Using cartridges filled with different MOF/COF inks, multiple layers of MOFs/COFs and mixed layers of different materials are also possible.^51^ Moreover, by precisely controlling where MOF/COF materials are deposited onto a substrate, a highly structured pattern can be generated. This is essential for integrated device fabrication, electronics, sensors, and microfluidic devices, where precise alignment and integration of multiple materials are crucial.^47,52^

Direct patterning techniques offer an attractive approach to precisely control the positioning and lateral structuring of the MOF and/or the COF pattern at micro- and nanoscale resolutions. Techniques such as soft lithography,^23,53^ pen-type lithography,^1^ photolithography,^54,55^ deep X-ray lithography, and electron-beam lithography^56^ enable high-resolution patterning by using masks, molds, or focused beams. Beyond lithography, other advanced printing methods such as 3D printing,^57−59^ aerosol printing,^60^ and inkjet printing^53,61^ have been introduced for more flexible and additive manufacturing of framework materials. These approaches provide material-efficient ways to create structured films without the need for physical molds or etching. Despite these advantages, direct patterning techniques present certain limitations. High-resolution lithographic techniques, for instance, require specialized equipment and multiple processing steps, leading to high operational costs and extended processing times. These methods also frequently generate chemical waste and involve solvents, contributing to environmental concerns. Moreover, techniques like electron-beam lithography and photolithography are often limited to small areas, which hinders their scalability and compatibility with larger substrates.^39^ Among the printing techniques, inkjet printing stands out as a noncontact method with high control over the formation of picoliter volume droplets that can be deposited with superior print resolution. An inkjet printer is able to generate these individual droplets at a high speed, only taking tens of milliseconds per droplet.^62^ The high speed in combination with the low volume and precise control on positioning provide a strong advantage toward reactive inkjet printing where different components can be mixed on the required spot.^63^ The reaction time after mixing of the components depends on the chosen chemicals, droplet size, and vapor pressure of the liquid, varying from seconds to several minutes.^47,64^ This high control over the individual droplets does reduce the overall speed on research based inkjet printers to between 0.01 and 0.1 m/s in comparison to alternatives such as spray coating (0.1 to 1 m/s). However, the film formation is of a lower quality in regards to film roughness, thickness (0.5 μm), and minimum width (50 μm) for spray coating.^65^ Aerosol printing, on the other hand, is able to produce much smaller droplets of 1–5 fL, resulting in spots of 5 μm, and allows for use of a large range of materials with viscosities between 1 and 2500 mPa·s, but this will result in a maximum printing speed of 5 mm/s, a minimum line thickness of 100 nm, and a width of 5 μm, whereas inkjet printing on a commercial scale can reach a minimum line width of 2 μm, a line thickness of 5 nm, and printing speeds of 5 m/s.^66,67^ Aerosol printing requires an additional carrier gas and needs to atomize the ink solution before it is able to print, making the application toward MOF/COF inks in direct printing complicated, as it might change the source material. All in all, inkjet printing is simple, mask-free, and of an additive nature,^68^ resulting in a cost-effective and rapid method for the production of complex patterns that are easily generated with computer-assisted information.^69−71^ Despite its advantages, inkjet printing of MOFs/COFs faces challenges in ink development, quality control, and equipment costs. While affordable office inkjet printers cost a few hundred euros, advanced R&D models with specialized features can cost hundreds of thousands of euros. Ink development requires stringent control over properties like particle size to prevent nozzle clogging, along with adherence to appropriate physicochemical properties of inks (viscosity, surface tension, and evaporation speed), which complicates solvent and additive selection. Additionally, achieving uniform thickness for MOF/COF films often requires multiple layers, increasing the risk of defects and affecting film uniformity. Drying-induced stresses during solvent evaporation can lead to cracking, while the relatively low throughput of inkjet printing limits its scalability for large-scale manufacturing compared to faster methods like roll-to-roll or spray coating. Furthermore, characterizations of printed MOF/COF (e.g., porosity, crystallinity, adhesions) are also desired while remaining challenging. These challenges highlight the need for optimization to fully leverage inkjet printing for MOF/COF applications.^36,72,73^

The patterning capability of inkjet printing for MOFs and COFs allows the creation of complex designs with varied adsorption, catalytic, and sensing properties. These functional differences depend on the composition and properties of the materials, which can be finely tuned across the patterned films.^24,39,74^ Applications of inkjet printed MOFs are found in various fields such as iodine sensing,^75,76^ ammonia sensing,^47^ aniline sensing,^77^ nitrite sensing,^78^ anticounterfeiting,^76,79^ information encryption,^80^ electrocatalysis,^78^ and *in vitro* diagnostic devices for cancer cell capture and detection.^81^

This work contributes significantly to the advancement of inkjet printing as a versatile and efficient method for producing MOF and COF films/patterns with tailored properties. Although only a few studies of reactive inkjet printing of COFs have been reported, we still include them in this review due to the similarities between MOFs and COFs during synthesis and activation procedures and the similar challenges when employing this technique. For reactive inkjet printing, both of them can be formed and activated under comparable conditions via printing building block inks, while achieving target structures with good crystallinity might require additional research efforts. In terms of direct inkjet printing, similar issues are also encountered, such as particle size control and ink stabilization. This review delves deeply into the printing procedure, ink formulation, and substrate considerations to optimize the process and attain high-quality MOF/COF films/patterns. The review also provides extensive insights into various ink formulations developed for MOF inkjet printing, encompassing considerations such as the selection of MOF particles, solvents, and additives. Moreover, it thoroughly explores essential printed MOF/COF properties such as porosity, physical-mechanical stability, film thickness and uniformity, and functionalization of the printed MOF/COF film. While 3D printing of these materials has been comprehensively reviewed in the literature,^82−86^ this is not the case for 2D or inkjet printing of framework materials. There are review articles on various aspects of inkjet printing technology, including (i) ink formulation of metal nanoparticles and complexes for printed electronics,^87^ (ii) inkjet printing of metal oxide-based precursors,^87,88^ (iii) of heterogeneous catalysis,^89^ and (iv) of lanthanide-organic frameworks for anticounterfeiting applications.^76^ However, to the best of our knowledge, there are no dedicated review articles specifically focused on the inkjet printing of MOFs or COFs.

## 2 MOF and COF Inkjet Printing Method

Inkjet printing stands out as a contactless technique for transferring ink to the substrate without applying physical contact between the ink dispenser and the substrate. Once the ink is prepared, the ink solution is loaded into the cartridge and the cartridge is then inserted into the inkjet printer. The ink solution flows from the cartridge to the nozzle head, from which it is ejected in the form of picoliter droplets. The ejected ink droplets are precisely deposited on the substrate to create the desired films or patterns. This can be achieved by either moving the substrate or adjusting the print head’s position. Subsequently, the printed ink undergoes postprocessing, such as UV irradiation to form a solid structure, high-temperature treatments like thermal annealing, sintering, or calcination to eliminate solvents with a higher boiling point, enhance adhesion, and modify the material’s structure.^90^

Inkjet printers operate in two primary modes: continuous and drop-on-demand (DOD). Continuous inkjet printing involves releasing ink as a liquid jet from the nozzle. This jet undergoes Plateau–Rayleigh instability, driven by surface tension, causing it to break up into droplets; here, all droplets that are not part of the print are captured and recycled. This mode is commonly employed in high-speed operations such as textile printing and labeling. DOD technology, on the other hand, allows for precise control over droplet size and placement, minimizing ink wastage. Within DOD technology droplets can be formed using either thermal or piezoelectric inkjeting, respectively, using heat or piezoelectric crystals to eject the droplets from the nozzle.^72,75,80,91^ The selection of the appropriate inkjet technology depends on factors such as ink properties, printing requirements, and desired resolution.^92^

**Scheme 1:**
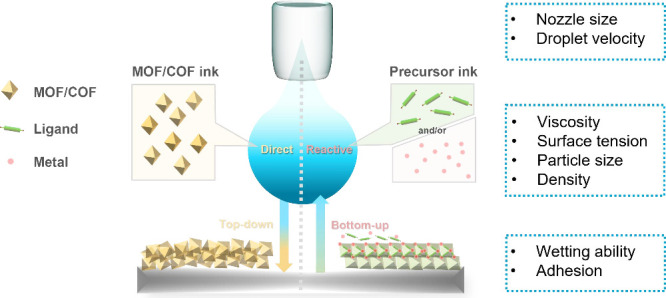
Schematic Illustration of Direct Inkjet Printing of a Presynthesized MOF/COF and Reactive Inkjet Printing of Precursor Solutions of a MOF/COF on Substrates and the Different Factors Influencing the Outcome of the Printing Process

For MOFs and COFs, the printing is typically performed using the DOD method, in which two distinct approaches have been developed by using different feed materials (Scheme 1). The first approach, known as direct inkjet printing, which has been used with MOFs but not with COFs so far, involves directly using a colloidal MOF solution as the ink for printing. The second approach, called reactive inkjet printing of MOFs/COFs, has the precursors dissolved in the ink, which, after being printed onto the substrate, undergoes a reactive process induced by e.g., curing or postprocessing to form the MOF or COF. This latter technique involves the printing of a single MOF/COF precursor solution or separate ink solutions of the different building blocks. A particularly elegant methodology was reported by Teo et al.: by adjusting the printer so that the drops from the two different monomer solutions coalesce in-air. This leads to more control over the printer geometry, and issues associated with directly printing a single ink containing the different precursors, like having the monomers being transported by Marangoni flow caused by the drying of solvents before the COF is formed, are circumvented.^61^ Postprocessing or curing may involve washing steps,^61^ drying steps at elevated temperature and/or vacuum,^39,61^ or be absent.^53^

In contrast to direct inkjet printing, reactive inkjet printing enables the convenient adjustment of reactant stoichiometry, synthesis conditions, and composition during this printing process, which is not possible with solutions of preformed materials. This precise approach facilitates the synthesis and patterning of MOFs and COFs onto substrates, and even the creation of gradients where one MOF gradually transforms into another.^64^

That being said, it is more prevalent to employ direct inkjet printing for various materials, where a well-dispersed suspension of the presynthesized material is directly utilized for inkjet printing.^78,89^ Reactive inkjet printing indeed requires careful material-specific optimization of the ink(s), printing conditions, and postprinting treatment. Direct inkjet printing allows for printing of a larger variety of MOFs/COFs, regardless of what synthesis conditions they require, and more generalized combinations of ink formulations, printing conditions, and substrates that can be used for a series of MOFs/COFs potentially can be successfully extrapolated to other MOFs/COFs with limited modification.^75,77−79,81,93−95^ Nevertheless, whether by employing a direct or reactive approach to inkjet print MOF and COF materials, understanding crystallization mechanisms should be underscored, as this might affect the inkjet printing process (e.g., controlling particle size plays an important role in smooth direct inkjet printing) and the final products (e.g., crystal growth procedures influence whether target structures with good crystallinity can be obtained in reactive printing).

The current review aims to delve into the optimization of the printing process, ink formulation, and substrate to achieve uniform printed layers and patterns. Table 1 provides a comprehensive overview of all published MOF/COF ink formulations, whether direct or reactive inkjet printing, their reported properties and applications, and the inkjet printers and sizes of nozzles being utilized.

**Table 1:** Inkjet Printed MOF/COF Overview^a^

Method	Material	Building block	Inkjet printer	Nozzle size	Particle size	Solvent	Additive	MOF/precursor concentration	Ink viscosity (mPa·s)	Ink surface tension (mN/m)	Substrate	Film/pattern	Application	MOF/COF property	ref
Direct	ZIF-70	Zn^2+^; nIM	MicroFab Jetlab-II system	80 μm		IPA					Interdigitated electrodes	Film	I_2_ vapor sensor	Mass	(75)
Direct	Ln-CPMAs	Eu^3+^, Sm^3+^, and/or Tb^3+^; H_2_DCPA	–	–	148 nm	EtOH		0.3 mg/mL			Paper	Pattern	Information encryption	Fluorescence	(93)
Direct	BMOF	Zn^2+^; PBA	HP DeskJet 3720	–	50 nm	Water		1.2 mg/mL			Filter paper and PVDF film	Film	Tumor cell capture and release	Fluorescence	(81)
Direct	Ln-BTC	Tb^3+^, Eu^3+^, and/or Sm^3+^; H_3_BTC	–	–	45 μm, 0.5–0.5 μm	EtOH, glycol, glycerin and DEG	SDS	about 2 mg/mL			Filter paper	Pattern	Information encryption	Fluorescence	(94)
Direct	MOF-5	Zn^2+^; H_2_BDC	GIX II Microplotter	–	1.5, 1.5, 1.5 μm	Deionized water		10 mg/mL			Microcantilever top-surface	Film	Aniline vapor sensing	Mass	(77)
Direct	HKUST-1	Cu^2+^; H_3_BTC		20 μm		DMSO, EtOH, and EG					Microbeam top-surface (Ni)	Film	Water vapor sensing	Mass	(95)
Direct	MOF-525	Zr^4+^; H_4_TCPP	MicroFab JetLab4 system	50 μm	100–700 nm	DMF		10 mg/mL			ITO glass	Film	Nitrite sensing	Conductivity	(78)
Direct	ZIF-8	Zn^2+^; 2-MI	GIX II Microplotter	–	70 nm	Water		10 mg/mL			Microcantilever top surface	Film	Competitive adsorption analysis	Mass	(96)
Direct	Ln-MOF	Eu^3+^, Tb^3+^, Gd^3+^, or Nd^3+^; BHC	Cannon Pixma MP495	–	0.5–8 μm	Water and organic solvents			0.83 (25 °C), 0.74 (40 °C)		PET foil and vegetal paper	Pattern	Information encryption	Fluorescence	(79)
Reactive	Pb-MOF	Pb^2+^; H_3_BTC	HP Desk Jet 2123	–	N.A.	DMSO, EtOH, and EG					Parchment paper and PET foil	Pattern	Information encryption	Fluorescence	(80)
Reactive	Cr-MIL-101	Cr^3+^; H_2_BDC	Canon IP7270	–	N.A.	DMSO, EtOH, and EG			5.02	30.09		Pattern			(47)
Reactive	HKUST-1	Cu^2+^; H_3_BTC	Canon IP7270	–	N.A.	DMSO, EtOH, and EG			5.76	33.49	Paper and flexible PET	Pattern			(47)
Reactive	Fe-MIL-101	Fe^3+^; H_2_BDC	Canon IP7270	–	N.A.	DMSO, EtOH, and EG			5.10	31.76	A4 paper, transparent and opaque PET foil	Pattern			(47)
Reactive	Co-MOF-71	Co^2+^; H_2_BDC	Canon IP7270	–	N.A.	DMSO, EtOH, and EG			4.33	31.34	A4 paper and filter paper	Pattern			(47)
Reactive	Mn-BDC	Mn^2+^; H_2_BDC	Canon IP7270	–	N.A.	DMSO, EtOH, and EG			5.11	32.1	Transparent PET and paper	Pattern and film	NH_3_ vapor sensing	Color	(47)
Reactive	Ni-BDC	Ni^2+^; H_2_BDC	Canon IP7270	–	N.A.	DMSO, EtOH and EG			3.61	30.33		Pattern			(47)
Reactive	Tb-BDC	Tb^3+^; H_2_BDC	Canon IP7270	–	N.A.	DMSO, EtOH, and EG					Transparent PET and paper	Pattern and film	NH_3_ vapor sensing	Fluorescence	(47)
Reactive	HKUST-1	Cu^2+^; H_3_BTC	JetLab system (MicroFab, Plano, and TX)	60 μm	N.A.	DMF; DMF		0.25 M;0.25 M	1.41;1.71	37.29;37.79	glass	Film	Selective adsorption of dyes	Porosity	(64)
Reactive	Cu(BDC)	Cu^2+^; H_2_BDC	JetLab system (MicroFab, Plano, and TX)	60 μm	N.A.	DMF; DMF		0.25 M; 0.25 M			glass	Pattern and film	Selective adsorption of dyes	Porosity	(64)
Reactive	Cu(ABDC)	Cu^2+^; H_2_ABDC	JetLab system (MicroFab, Plano, and TX)	60 μm	N.A.	DMF; DMF		0.25 M; 0.25 M			glass	Pattern and film	Selective adsorption of dyes	Porosity	(64)
Reactive	Zn(BIM)_2_	Zn^2+^; HBIM	JetLab system (MicroFab, Plano, and TX)	60 μm	N.A.	DMF; DMF		0.25 M; 0.5 M			glass	Pattern and film			(64)
Reactive	Co(BIM)_2_	Co^2+^; HBIM	JetLab system (MicroFab, Plano, and TX)	60 μm	N.A.	DMF; DMF		0.25 M; 0.5 M			glass	Pattern and film			(64)
Reactive	Cyt cZIF8	Zn^2+^; 2-MI; Protein	Epson ME-10	–	N.A.	DMSO, EtOH, EG; DMSO, EtOH, EG; Water, glycerol	−; −; TrionX-100	125 mmol/L; 1 mol/L; 4 mg/mL	2.8; 2.8; 2.80	36.00; 36.00; 38.00	Filter paper, PVC film, PET film, and hydrophilic printing film.	Pattern	H_2_O_2_ sensing	Color	(97)
Reactive	HKUST-1	Cu^2+^; H_3_BTC	Hewlett-Packard Officejet 6000	–	N.A.	DMSO, EtOH, and EG					PET foil, paper, and textile	Pattern and film	HCl, NH_3_, H_2_S, Cl_2_, and CO_2_ vapor sensing	Color and mass	(39)
Reactive	HKUST-1	Cu^2+^; H_3_BTC; NaOAc	Custom-built inkjet printer	20 μm	N.A.	N.A.; EtOH and DI water; EtOH and DI water		−; 10 mM; 60 mM	N.A.; about 2.6; −	N.A.; about 27; −	Cu^2+^ exchanged TCNF film.	Pattern	Information encryption	Fluorescence	(98)
Reactive	[Zn_2_(adc)_2_ (dabco)_2_]	Zn^2+^, DABCO and ADC	Hewlett-Packard Officejet 6000	–	N.A.					Paper	Pattern			Fluorenscence	(39)
Reactive	RT- COF-1	TAPB; BTCA	Fujifilm Dimatrix (DMP-2831)	21 μm	N.A.	DMSO	1 mg/mL; 0.43 mg/mL			Acetatepaper; SiO_2_	Pattern				(53)
Reactive	RT- COF-1	TAPB; BTCA	Custom-built inkjet printer	60 μm	N.A.	Glacial acetic acid	14 mg/mL; 6.45 mg/mL	1.21; 1.25	37; 38	Glass; Paper	Pattern			Fluorenscence	(61)

a Abbreviations: nIM, 2-nitroimidazole; H_2_DCPA, 4,5-dichlorophthalic acid; PBA, 1*H*-pyrazole-3-boronic acid; H_3_BTC, 1,3,5-benzenetricarboxylic acid; H_2_BDC, 1,4-benzenedicarboxylic acid; H_4_TCPP, *meso*-tetra(4-carboxyphenyl)porphine; 2-MI, 2-methylimidazole; BHC, benzenehexacarboxylic acid; H_2_ABDC, 2-amino-1,4-benzenedicarboxylic acid; HBIM, benzimidazole; DABCO, 1,4-diazabicyclo[2.2.2]octane; ADC, 9,10-anthracenedicarboxylate; TAPB, 1,3,5-tris(4-aminophenyl)benzene; BTCA, 1,3,5-benzenetricarbaldehyde;

## 3 Optimization of Inkjet Printing of MOFs and COFs

The production of MOF or COF films and patterns through inkjet printing is a complex process influenced by multiple factors, including the print procedure, ink formulation, and substrate. These elements collectively shape the quality and characteristics of the resulting MOF/COF layer. Challenges regarding inkjet printing MOFs/COFs require a good understanding and study of the interplays among each component (namely, MOF/COF particles (or building blocks), solvents, inkjet printers, and substrates). For instance, when using inkjet printers, one first needs to be aware of the hardware parameters and proper operation steps (Section 3.1). Furthermore, ink printability and compatibility are key considerations. That means controlling MOF/COF ink properties, particularly particle size in direct inkjet printing, to prevent clogging of the printer nozzles (Section 3.2.1). Additionally, MOF/COF inks need to have appropriate physicochemical properties to be in the printable range and colloidally stable, which restricts the selection of solvents and additives and makes the ink formulation more complex (Sections 3.2.2 and 3.2.3). Meanwhile, on the receiving substrate side, the intricate interactions among particles (or building blocks), solvent components and substrate would affect the printed MOF/COF quality (Section 3.3). When using RIJ, obtaining the target MOF/COF structures with desired properties (e.g., crystallinity, porosity) requires research efforts (Sections 4.1 and 4.3). The drying process poses another challenge, as solvent evaporation can create drying-induced stresses, resulting in coffee ring effects, cracking, or other defects in the printed MOF/COF (Sections 3.3 and 4.3). Last but not least, controlling the thickness of the deposited layer involves printing multiple layers. This layering process can introduce defects and affect uniformity (Section 4.4). These limitations highlight the need for careful optimization and present areas for future development to fully harness inkjet printing potential in MOF/COF applications. A deeper understanding of these factors allows for successful inkjet printing of MOFs or COFs. The focus of this paper is to investigate and comprehend the effects of these factors in order to attain uniform and well-defined MOF or COF films and patterns with the desired properties.

### 3.1 Inkjet Printer Process Optimization

The selection of the right hardware is a critical factor that impacts the resolution, precision, and deposition uniformity of MOF ink droplets during fabrication.^91^ Commonly used inkjet printers for printing MOFs and COFs include research inkjet printers (e.g., MicroFab jet Lab,^64,75,78^ GIX II Microplotter,^77,96^ Süss Microtec PixDro LP50,^25^ and Fujifilm Dimatrix)^53^ and office inkjet printers (e.g., HP DeskJet 3720,^81^ HP Desk Jet 2123,^80^ Canon IP7270,^47^ and Epson ME-10^97^).

To maintain consistent and reliable printing performance, first the MOF/COF ink has to be a stable dispersion with particle sizes at least 50 times smaller than the nozzle openings to avoid clogging or blocking of the nozzle^78,99^ (Section 3.2). If this is not the case, in between printing, the printhead and ink reservoir need maintenance. During printing, regular purging (i.e., pushing ink through the nozzle using overpressure) and wiping of the printhead are advised. For longer timeframes (e.g., several hours), in between printing steps, spitting could be used. During spitting, the nozzles of the printhead are activated to generate a chosen number of droplets to prevent drying of the ink within the printhead. For longer intervals between printing, or when changing ink, thorough cleaning of the printhead and ink reservoir is required. Cleaning should be done with similar solvents from the COF/MOF ink first by washing the ink reservoir, followed by purging the printhead from the clean reservoir with the same solvents. After cleaning, the ink reservoir and printhead should be carefully air-dried before being refilled and reused.^76,80^ Use of incompatible solvents should be avoided to prevent miscibility issues during cleaning.

By selecting the right nozzle size, with typical diameters between 30 and 100 μm, the print resolution, quality, and speed are influenced.^100^ The nozzle size limits the minimum droplet volume, while smaller nozzle sizes are associated with higher resolution and finer details of the printed pattern. However, it is important to note that smaller nozzle sizes may also result in lower printing speeds due to the reduced volume of ink ejected per nozzle.^89^ By changing the printer settings, such as the waveform applied to the nozzle, it is possible to optimize the droplet formation, volume, and velocity.^76,80^ This, in combination with the printing speed and drop spacing, determines the resolution of the print. The layer uniformity will depend on the interaction of the MOF ink with the substrate where the substrate surface can be modified to improve the layer formation; however, the final print quality will strongly depend on a smooth printing and on avoiding nozzle clogging.^101^ Therefore, it is necessary to optimize the composition of the MOF/COF ink, as will be discussed in Section 3.2. After printing one or several postprocessing steps might be required, which might include passive drying to the environment, active drying at a higher temperature, or curing by UV irradiation. Subsequently, a solvent development step could be implemented by immersing the substrate into a suitable solvent to remove any residual primal solvents, followed by a final drying step.^47,80^

For the reactive printing approach, smaller picoliter volume droplets are preferred, as they show an improved mixing rate and heat transfer during the MOF/COF formation, resulting in lower material consumption during the formation of the MOF/COF layers.^64^

### 3.2 Ink Formulation

The ink formulation is another critical aspect that affects the procedures. On the print head side, formulations impact the ink stability, printability, and compatibility with the printer components. On the receiving side (substrate), formulations determine the final resolution, smoothness, continuity, and uniformity of the printed MOF/COF materials. A successful MOF/COF formulation should meet the requirements of both sides.^102,103^ However, most of the MOF/COF inks that have been developed so far are based on trial and error and lack a fundamental understanding. The composition and properties of the ink are significantly influenced by particle size, particle/precursor concentration, solvent(s), solvent evaporation rate(s), and any additives present. Subsequently, the influence of each component in the ink will be introduced in detail.

#### 3.2.1 Particles

Direct inkjet printing presents challenges related to particle sedimentation and dispersion during the ink formulation process. According to Stokes’ law, the terminal velocity or sedimentation rate is proportional to the square of the particle diameter. To prevent clogging, it is preferred to use smaller primary particles with slower sedimentation rates in the ink formulation.^104^ To ensure ink stability and prevent clogging during printing, sedimentation tests are ideally conducted to assess the dispersibility of MOF particles.^78,105^

**Figure 1:**
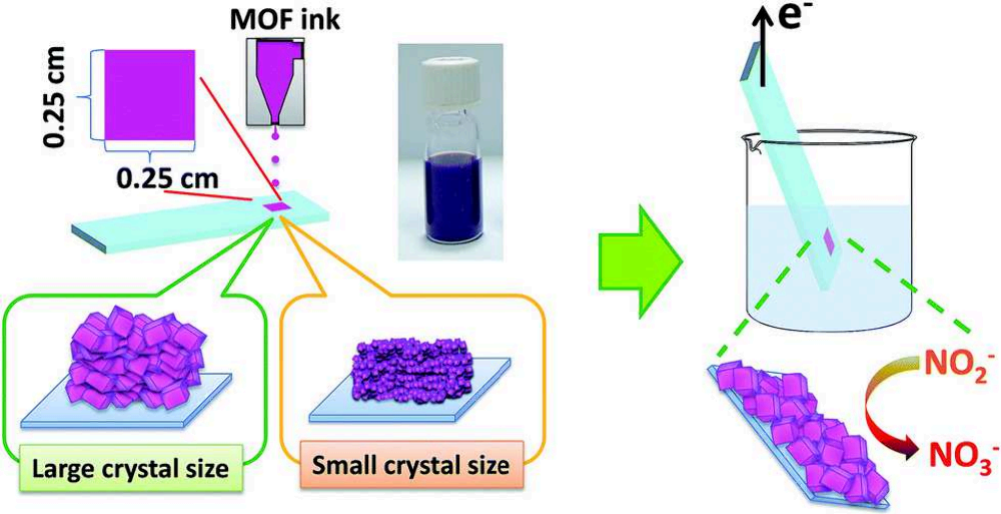
Denser film can be obtained via inkjet printing smaller crystals. Reproduced with permission from ref (78). Copyright 2016 Royal Society of Chemistry.

The size of the MOFs/COFs thus plays a critical role in inkjet printing, particularly for achieving high-resolution patterns. To ensure smooth printing and avoid nozzle clogging, it is necessary for the particles in the ink to be equal to or smaller than 1/50 of the nozzle diameter.^78,99^ It is also preferable to use particle sizes below 500 nm to optimize printing quality and precision, and for applications requiring even higher resolution of printed pattern, sizes smaller than 200 nm are recommended.^106^ Also, the crystal sizes strongly affect the microstructure of printed MOF thin layers since smaller crystal sizes form denser and thinner films with increased contact areas to the substrate. Indeed, MOF-525 particles with a smaller crystal size were found to form a thinner and denser thin film with a larger contact area with the substrate, which improved the charge transport between the thin film and underlying conductive substrate (Figure 1).^78^

It is important to note that nanoparticles dispersed in ink solutions tend to form secondary agglomerates on a micrometer scale, which can result in particle sedimentation. To address this, it is important to manage and minimize agglomeration by stabilizing the inkjet solution using dispersing agents^89^ or optimizing the composition of solvents to form a stable MOF/COF colloidal system. We will subsequently discuss strategies to obtain MOF and COF particles of appropriate nanosize and how these may be stabilized in suspensions, ending with some considerations regarding particles in the context of reactive inkjet printing.

##### 3.2.1.1 Particle Size Control

There are two general approaches to attain colloidal MOFs and COFs with a controlled size: bottom-up, in which the framework materials are synthesized in a way that limits crystal growth and prevents aggregation, and top-down, in which COFs and MOFs are postsynthetically reduced to smaller sizes. We first discuss the bottom-up approach.

Utilizing bottom-up approaches to regulate MOF/COF particle sizes highlighted the importance of understanding the crystallization mechanisms for the two materials. Classical nucleation theory (CNT) has been employed as the basis to understand crystal growth, where the model divides crystallization into two periods, nucleation and crystal growth. However, most MOF growth follows nonclassical nucleation models, which means an intermediate phase (so-called metaphase) having a local energy minima can form between the nucleation and crystal growth.^107^ For MOFs, there are several methods available to synthesize them bottom-up in smaller sizes with a narrow size distribution. Each depends on the many intrinsic and extrinsic synthetic parameters that can be adjusted to manipulate the MOF crystal growth. Intrinsic factors affecting MOF formation include precursor concentration and reaction parameters, such as temperature, solvent composition, and reaction time during the synthesis process.^89^ By carefully optimizing these parameters, it is possible to control the nucleation and crystal growth processes, leading to the formation of smaller MOFs. Extrinsic factors like modulators, microwave irradiation, and ultrasonication during synthesis can affect the nucleation and growth. The last two techniques provide more efficient and uniform heating and promote cavitation and acoustic effects, respectively, resulting in smaller and more uniform MOF sizes.^5,108,109^ In terms of COF growth, it often relies on reversible reactions, such as imine condensation, to enable defect repair and ensure crystallinity, which can be more demanding compared to typical MOF synthesis methods.^110^ To achieve ordered and crystalline structures in COFs, specific synthetic methods, such as solvothermal conditions, are often necessary to facilitate the dynamic reversible covalent reactions required for defect correction and crystal formation.^110^ Through these conditions, defects can be repaired and the thermodynamic product (in contrast to the kinetic product under nondynamic conditions) is formed, which can be very crystalline.^110^ This way, 2D ordering is first achieved within the sheets, followed by 3D ordering between the sheets to get the final crystalline product.^111^

In general, controlling the particle size for COFs poses more of a challenge, as the COF particles tend to aggregate in the synthesis process. As said, in the bottom-up approach, COFs are synthesized under such conditions that they have a stable and uniform particle size and do not aggregate. So far, this has been shown only for 2D COFs, but it is expected that 3D COF colloids can be made with a similar procedure. One such example is that by selecting the right experimental conditions (in this case, a room temperature synthesis in acetone with acetic acid as a catalyst), monodisperse amorphous imine COF nanospheres could be obtained.^112^ By then changing the conditions under which the reversible imine-bond formation occurs, the obtained nanospheres can be crystallized, also increasing their surface area.^113^ As the first step is compatible with nanoparticle chemistry, this method can be used to encapsulate nanoparticles or create a core–shell particle with a COF shell. This way, the COF nanoparticles can be given additional properties, such as being magnetic^114^ or catalytically active,^113^ by choosing nanoparticles with the desired properties to encapsulate.

With regard to COFs, a very promising route is the use of acetonitrile and other nitrile-containing compounds as (co)solvents during synthesis, as they can prevent aggregation to keep the COF particles in an initial colloidal state with colloid sizes of 30 nm. These can then be grown into larger sizes by slow monomer addition.^115,116^ Two other parameters by which the colloid size can be controlled are the initial building block concentration^117^ and, for imine-based COF colloids, the concentration of acetic acid.^118^

Another bottom-up method to create colloidal MOF/COF is the use of interfacial synthesis routes. In these routes, materials are synthesized at the interface between two different phases. During the interfacial synthesis, the rate is diffusion limited, creating stable and crystalline MOF/COF films under conditions which would normally not yield crystalline MOFs/COFs.^119,120^ For MOFs, nanosheets can be obtained via liquid/liquid, liquid/air, and liquid/solid interfaces.^121^

For liquid/liquid interfacial synthesis, metal ions and ligands are dissolved into two immiscible liquids, respectively. Through adjusting the concentration of ligands, the thickness of sheets can be adjusted, yet the thickness of the MOF sheets obtained is normally over 100 nm. Liquid/air interfacial synthesis can be employed in preparing ultrathin or even single-layer MOF sheets. Using this method, a small quantity of organic solvent(s) containing ligands is dropped onto the water surface. After the evaporation of organic solvents, a liquid/air interface is formed, and the crystal growth takes place. Currently, most MOF sheets which have been obtained with this method are porphyrin- or triphenylene-based MOFs, and the thickness can be as low as a few nanometers.^122^

MOF sheets can also be synthesized at the liquid/solid interface by depositing organic ligands on metal/MOF surfaces. This actually opens up an opportunity for reactive inkjet printing. In current reactive inkjet printing, both metals and linkers are usually developed into precursor inks and then deposited onto substrates. However, this can also be achieved by depositing linkers onto metal-containing substrates directly. In this way, metal-containing surfaces can serve as substrates and a reactant simultaneously. This also can potentially increase the adhesion between the smooth metal surface and MOFs, and produce oriented films/patterns. In a recent study,^98^ this approach was utilized to produce a HKUST-1 film on Cu^2+^ containing a 2,2,6,6-tetramethylpiperidine-1-oxyl (TEMPO)-oxidized cellulose nanofiber (TCNF) substrate.

As for COFs, most studies have focused on liquid/liquid interfacial synthesis. If this two-phase system is vigorously stirred, then an emulsion is formed, and COF nanosheets with a controlled size between 200 and 600 nm can be synthesized. The nanosheet size is determined by the ratio of the two phases and the concentration of both building blocks.^123^

In contrast to the bottom-up approach, in the top-down approach, COFs and MOFs are synthesized under normal, aggregating conditions and reduced in size postsynthetically. After synthesis, mechanical and chemical pathways can also be employed to reduce the sizes of as-synthesized MOFs/COFs to a printable scale. Mechanical milling of MOF powders can be used to break down larger particles into smaller ones, regularly with a limited loss of porosity and crystallinity.^5,108,109^ Ultrasonication of a MOF suspension or solution is also commonly applied to disperse aggregates and promote size reduction.^75,124^

With regard to 2D COFs and MOFs, they are formed by two-dimensional sheets that stack on top of each other with weak interlayer interactions (e.g., π-π interactions, hydrogen bonding, van der Waals interactions) along the vertical direction. These larger structures aggregate further through significant intermolecular attractive forces.^115^ Reducing their size postsynthetically thus means overcoming or disrupting these interactions, a process called exfoliation, which results in individual sheets, which are often colloidal in size. Exfoliation has been proven to be an efficient method for the preparation of MOF/COF sheets, including ultrasonic, mechanical and chemical exfoliation.

Among all of the exfoliation methods, ultrasonication exfoliation is one of the most widely used approaches for fabricating 2D MOF/COF sheets. For COF sheets, this method has been used to create suspensions with COF nanosheets with sizes ranging between 200 and 600 nm and with a narrow size distribution,^106,125,126^ while for MOFs, even thinner nanosheets (between 1 and 250 nm) can be fabricated.^121,127^ Mechanical exfoliation was also utilized to obtain the MOF/COF nanosheets. For example, MOF sheets can be produced using the Scotch tape method^128^ and shaking exfoliation.^129^ COF sheets were obtained via mechanical delamination of as-synthesized COFs with a mortar and pestle to achieve single-layer covalent organic nanosheets. However, in mechanical exfoliation, there is no control over the lateral sizes of the nanosheets.^130^ Besides physical exfoliation, chemical methods are also employed to exfoliate the MOFs and COFs. For example, acid exfoliation was used to exfoliate COFs, in which the imine linkages are protonated, leading to electrostatic repulsion between the sheets.^131^ Another way to chemically exfoliate MOF/COF sheets is through postsynthetic modification. For example, an anthracene-based COF can undergo a Diels–Alder click reaction with a maleimide derivative to make any stacking sterically impossible.^132^ Ding and co-workers obtained MOF nanosheets through the intercalation/chemical exfoliation method.^133^ First, 4,4′-dipyridyl disulfide (DPDS) was inserted into the interlayer of Zn_2_(PdTCPP). Then DPDS was selectively reduced by trimethylphosphine (TMP), and then, isolated MOF nanosheets were obtained.

Often, the surface areas of the top-down created MOF/COF particles are not reported, but when they are, a significant loss of surface area is reported.^132^ The crystallinity is usually lower, as well. This could be because of the disordered stacking of the exfoliated sheets^131^ or (partial) destruction of the pores. In contrast, the previously discussed bottom-up method of synthesizing MOF/COF colloids gives control over the size of the colloids without sacrificing the crystallinity or porosity of the materials.^134^

**Figure 2:**
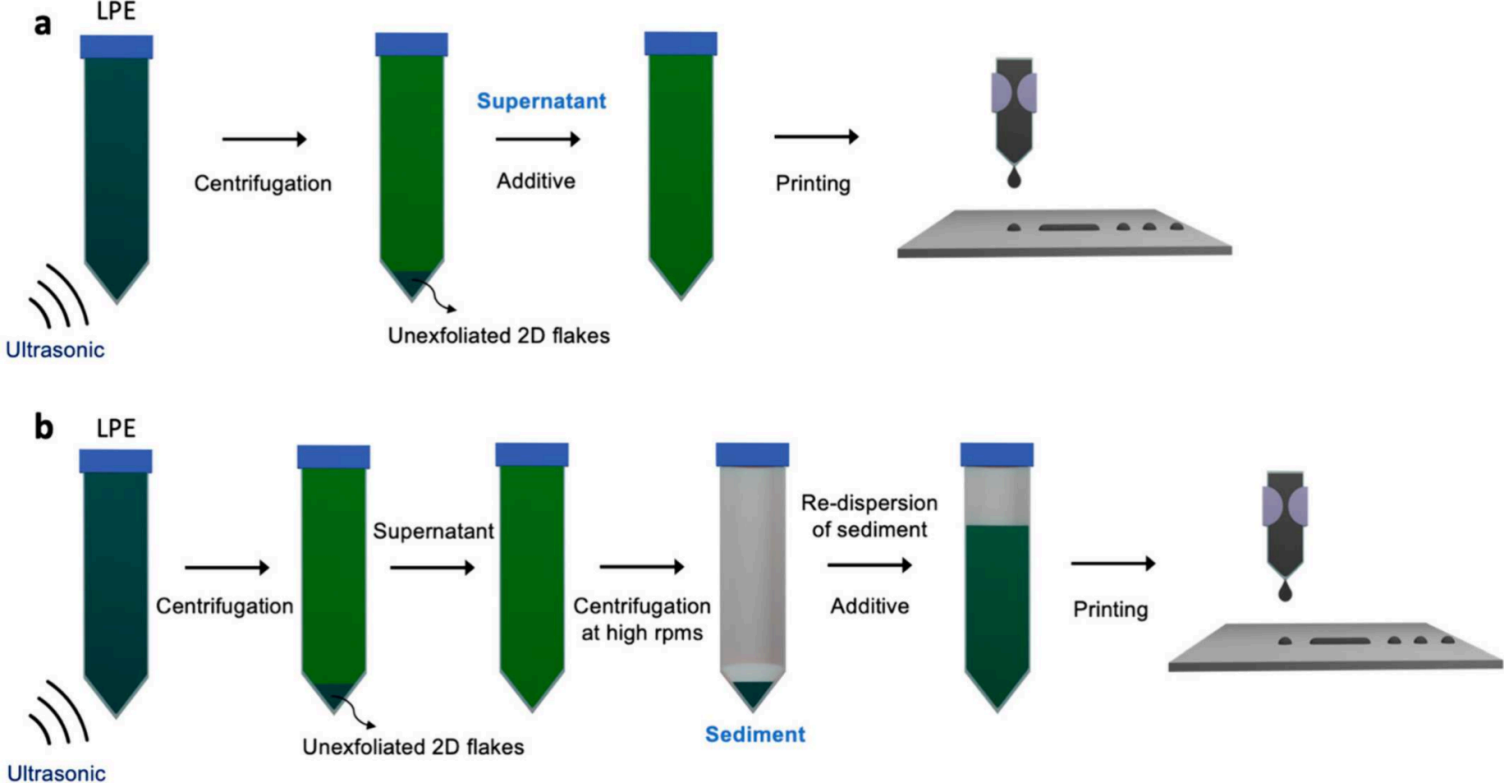
Preparing inks with exfoliation either by (a) direct ink formulation or (b) solvent exchange ink formulation. Reproduced from ref (135). Available under a CC-BY 4.0 license. Copyright 2021 by the authors. Licensee MDPI, Basel, Switzerland.

Exfoliation methods can produce nanosized materials that align with the size specifications for inkjet printers, making exfoliated materials potentially promising for producing MOF/COF inks. However, to the best of the authors’ knowledge, exfoliated MOF/COF inks have not been reported yet. Nevertheless, it is noteworthy that other exfoliated 2D materials have been successfully developed into inks and printed,^135^ such as graphene,^136^ MoS_2_,^137^ and black phosphorus (BP).^138^ Especially for liquid phase exfoliation (LPE), there are two ways to prepare inks, combined with exfoliation procedures (Figure 2). The first way is utilizing the supernatant of exfoliated materials as the ink directly when the supernatant satisfies the physico-chemical parameters for inkjet printing. Alternatively, the second way, which involves an additional solvent exchange step, can be adopted. Therefore, we believe that 2D MOF/COF sheet inks can also be prepared following this methodology.

Alternatively, one may also obtain MOF particles with a narrow size distribution and size control from a polydisperse synthesis via separation techniques such as membrane filtration and centrifugation. In membrane filtration, the MOF solution is passed through a membrane with a specific pore size, allowing smaller-sized MOF particles to pass through while retaining larger particles.^47,80,97^ Centrifugation, on the other hand, separates particles based on their sedimentation rates under high centrifugal forces.^139^ After centrifugation, the supernatant could be collected, containing MOF particles with a narrower size distribution. So far, this method has not been utilized on COFs, though in principle this methodology could be followed as well.

For more comprehensive information on these topics, we refer to research articles and reviews dedicated to the synthesis and size control of MOFs and COFs.^5,107−109,121,127^

##### 3.2.1.2 Colloidal Stabilization

Once MOF/COF particles of the desired range are obtained, it is key that they are colloidally stable in the solvent targeted for inkjet printing. In principle, extended Darjaguin–Landau–Verwey–Overbeek (exDLVO) theory can be used to understand the parameters to obtain a stable MOF/COF ink system. Yet, so far it is only sparingly used to describe MOF colloidal stability.^140,141^ The theory states that the total potential energy is the sum of the attraction potential and repulsion potential. When the absolute value of attraction force is greater than the repulsive forces, the particles aggregate and the colloidal system is unstable. In contrast, if the repulsive forces are stronger than the attractive forces, then an energy barrier could form, and then the colloidal solution would be well dispersed.

**Figure 3:**
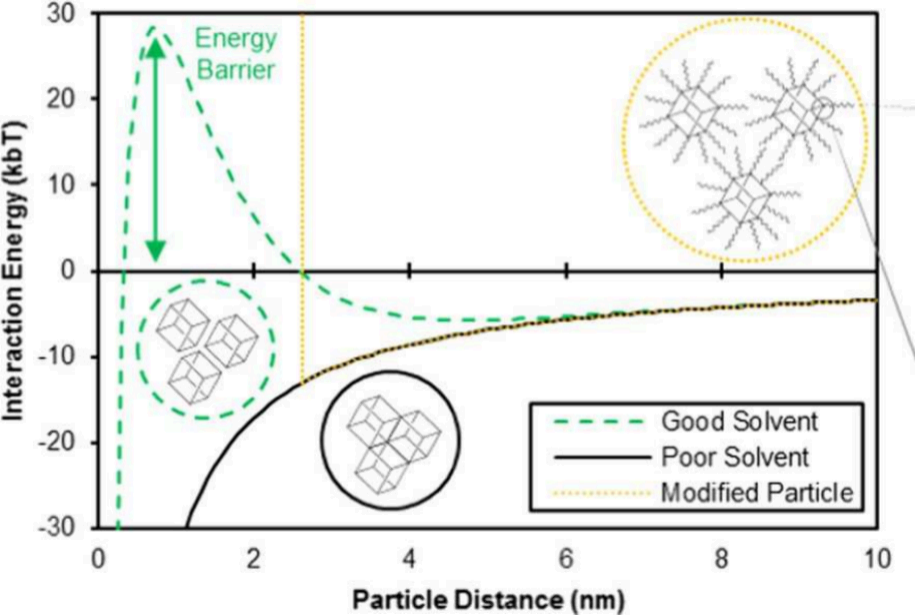
MIL-101 colloidal model based on DLVO theory: dashed green line, high-dielectric-constant solvent; solid black line, hydrophobic solvent; yellow dotted line, surface-modified MIL-101. Reproduced from ref (140). Copyright 2021 American Chemical Society.

Petit et al.^140^ were the first to quantify the surface properties parameters for MOF particles, namely MIL-101, such that the attractive and repulsive forces related to the extended DLVO theory could be estimated (Figure 3). They found that the electric double layer repulsive forces are high in high-dielectric-constant solvents (green dashed line in Figure 3), leading to stable suspensions, while they diminish in low-dielectric-constant solvents (black solid line in Figure 3), thus leading to unstable suspensions in hydrophobic solvents. They could induce colloidal stability also in hydrophobic solvents via functionalization of the external surface by hydrophobic molecules that induced steric repulsion (yellow dotted line in Figure 3).^140^ Later, similar estimations were attempted for ZIF-8 by Yang and Wen.^141^

Brozek et al.^142^ recently pointed out that in contrast to other types of nanoparticles, nano-MOFs very regularly do not need any surface functionalization with bulky ligands to achieve multiweek colloidal stability in polar solvents.^143−147^ They point out that electrostatic forces alone cannot explain colloidal stability. They showed for a series of eight different nano-MOFs that solvents that show a high solubility for the constituent MOF linkers, and where solvent and pore size allows for the solvent to be densely packed inside the pores, correlate with high colloidal stability. While the same general trend is observed that polar solvents stabilize MOF colloids, they found an example of a MOF with a linker with significant solubility in hydrophobic solvents, namely ZIF-11, for which a stable suspension of this MOF could be formed in toluene.^142^

As discussed previously under “particle size control” in Section 3.2.1, many COF colloidal solutions are particularly stable in nitrile-containing solutions. In fact, it has been shown that acetonitrile (and in some cases other nitrile-containing compounds such as butyronitrile, propionitrile, benzonitrile and pyridine)^148^ stabilize COF particles with boronate ester,^41,115,116^ boroxine,^148^ and imine^117,149^ linkages. These colloids have been reported to be stable for months,^117,148,149^ or even indefinitely.^41^ For boron-containing COFs, this stability is based on the interaction of the nitrile as a Lewis base with Lewis acid moieties in the COF (i.e., the boron sites). Factors that contribute to stability with regard to other linkages or of COF colloids in other solvents are not yet understood on a theoretical level.

COF colloids grown in acetonitrile have been used for several applications, such as porous liquids,^117^ photonic crystals,^118^ and, most relevant for this review, spray coating^41^ and aerosol jet printing.^60^

##### 3.2.1.3 Reactive Inkjet Printing

Utilizing an ink solution where the precursors are dissolved helps prevent potential particle sedimentation in reactive inkjet printing. However, the premixed procedure of precursors can introduce challenges in ink stability over extended storage periods and may also lead to print head clogging due to the deposition of preformed particles. To address these potential issues, separate ink solutions of building blocks were used for reactive inkjet printing. This allows precursors to only react and form the desired MOFs/COFs *in situ* after being printed on the substrate. For instance, by using separate inks containing ligands or metal ions dissolved in dimethylformamide (DMF), successful printing of five different MOFs, including Cu_3_(BTC)_2_, Cu(BDC), Cu(ABDC), Zn(BIM)_2_, and Co(BIM)_2_ (BTC, 1,3,5-benzenetricarboxylic acid; BDC, 1,4-benzenedicarboxylic acid; ABDC, 2-amino-1,4-benzenedicarboxylic acid; BIM, benzimidazole), has been achieved.^64^

When reactive inkjet printing is used, the MOFs/COFs that can be printed might be limited, especially excluding those that require harsh synthesis conditions. For example, in the work by Goel and co-workers,^47^ an attempt was made to synthesize some MOFs, which normally require high temperature/pressure synthesis conditions via reactive inkjet printing. It is found that the X-ray diffractograms of the obtained MOF films did not match well those of the targeted Cr-MIL-101, Fe-MIL-101, and Co-MOF-71 structures. In terms of COFs, reactive inkjet printing has been achieved with a specific imine-based COF that forms at room temperature.^53,61^ As the formation of COFs under mild conditions has been extensively investigated in light of green chemistry, this might expand the library of suitable COFs for reactive printing.

**Figure 4:**
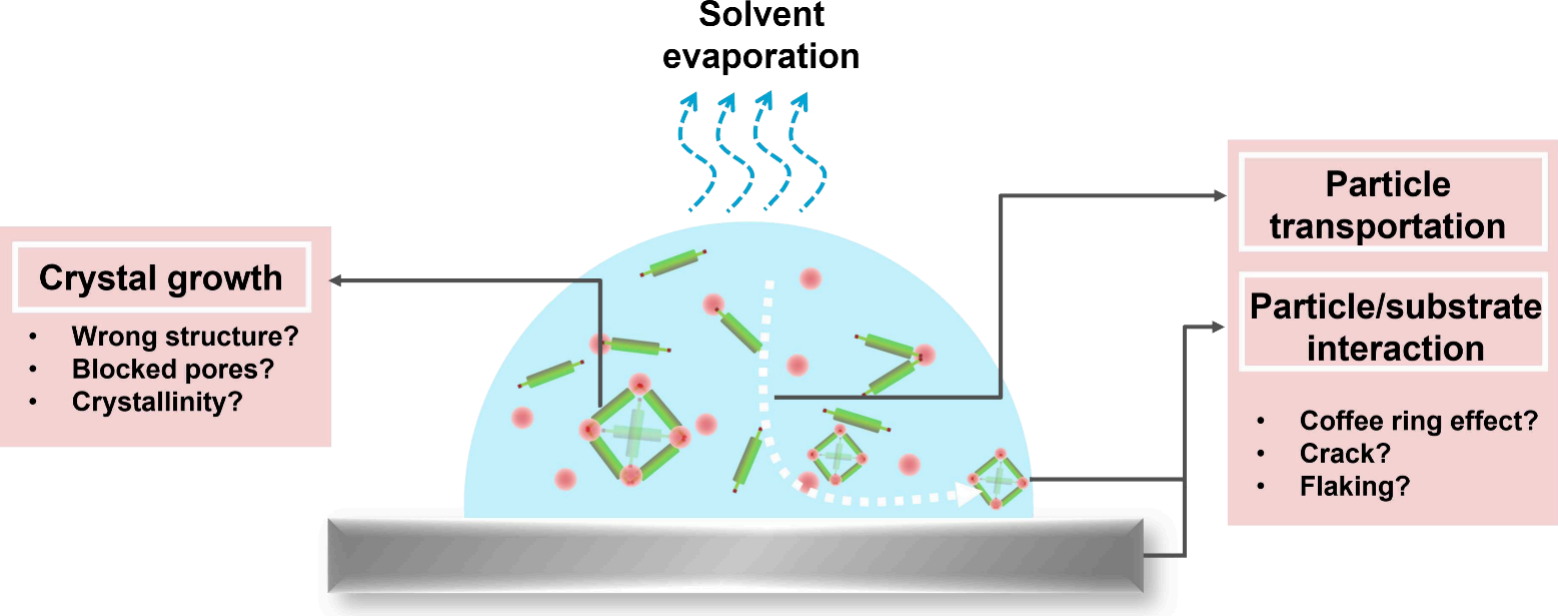
Challenges for reactive inkjet printing.

Additionally, reactive printing procedures are not mere depositions of precursors. Attention to the precise procedures after droplet deposition is critical because crystal growth, evaporation of solvents, and particle transportation will happen simultaneously, complicating the quality control of the MOF/COF films, as shown in Figure 4. Challenges with reactive inkjet printing include obtaining the target MOF/COF structures, decent crystallinity and/or porosity, avoiding the production of unknown phase structures, and forming a uniform and continuous MOF/COF film on the substrate. Crystallinity will be the main challenge in the reactive printing of COFs, as COFs firstform an amorphous polymer, which crystallizes through defect healing.^150,192^ This process may be more challenging when performed directly on a substrate.

#### 3.2.2 Solvents

When optimizing solvents for MOF/COF inks, the suitability of the solvent concerning the inkjet printing process and the interaction among solvents, MOF/COF particles and/or precursors, and the substrates should all be taken into consideration. Solvents form the main component of the ink solution and are responsible for dispersing the MOFs/COFs or the precursors as well as other additives. The selection of suitable solvents and additives is essential for achieving the desired stability, viscosity, and surface tension of the ink to fit the working region of the inkjet printers (vide infra). The viscosity of the ink affects the flow behavior and droplet formation, which influences the deposition accuracy and pattern fidelity. The surface tension of the ink also affects the droplet formation and, in addition to that, affects the wetting behavior and adhesion of the ink droplets to the substrate.^76,79^

Ink solutions with excessively high viscosity face challenges in smoothly releasing from the printer nozzle. On the other hand, low-viscosity inks tend to form unstable droplets during the printing process, resulting in the formation of satellite drops. Therefore, it is recommended to maintain a viscosity range of 1–25 mPa·s in DOD inkjet printers to ensure smooth release from the nozzle and avoid the formation of unstable droplets and satellite micro-drops. In addition, it is advised to maintain a surface tension within the range 20–50 mN/m in DOD inkjet printing. Higher surface tension values can hinder proper droplet formation, while lower values may lead to air ingestion and droplet dripping.^49,150^

**Figure 5:**
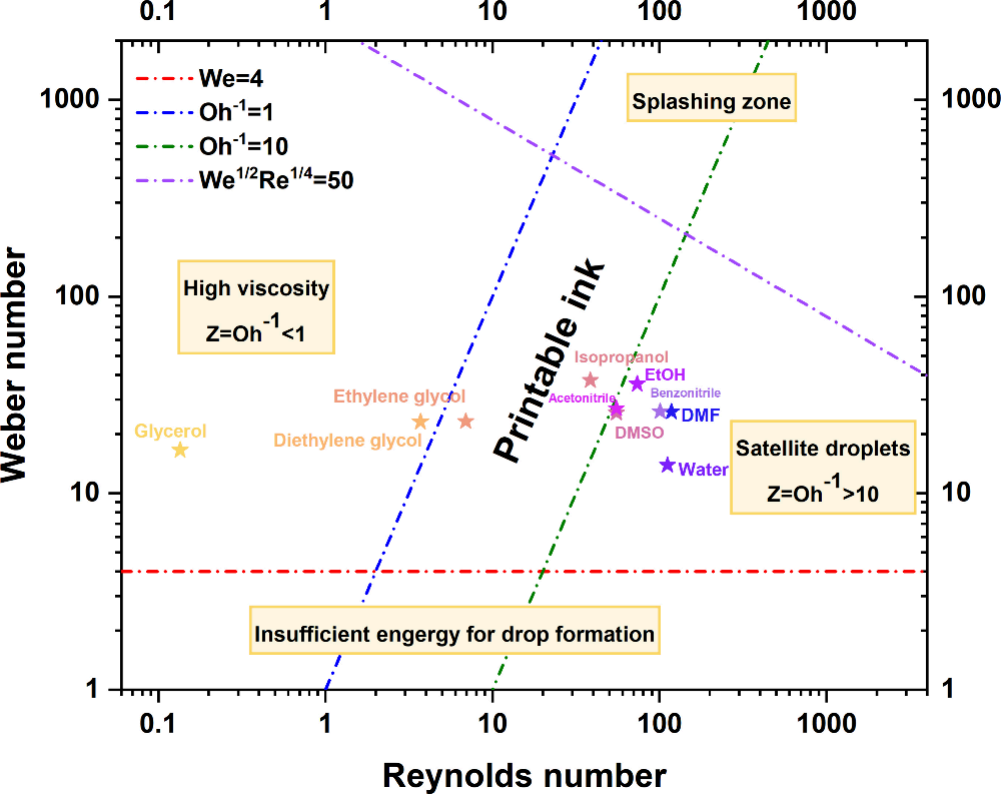
Ink printability region based on the Reynolds and Weber numbers. Solvent printability was added assuming the nozzle size (*D*) is 20 μm and the velocity of the droplet (*U*) is 10 m/s. Adapted with permission from ref (89). Copyright 2020 Royal Society of Chemistry.

In practice, droplet formation is a complex process of transferring the available energy supplied by the print head to the ink with regard to the creation of surface area, momentum, and velocity of an ink droplet. The surface tension (σ, mN/m), density (ρ, g/cm^3^), kinematic viscosity (ν, mPa·s), dynamic viscosity (η, m^2^/s), droplet radius (*R*, μm), and fluid drop velocity (*U*, m/s) are the most important parameters that govern this energy transfer. The above-stated working ranges of these parameters can also be combined to the dimensionless numbers like the Reynolds number (Re, eq 1) and the Weber number (We, eq 2) to represent the ink fluidic properties.^151,152^ To further simplify the analysis, a single parameter known as the inverse Ohnesorge number (*Z* = Oh^–1^, eq 3) is commonly used to describe the inkjet printing condition, which incorporates the Re and We numbers.^151^ As given in eq 3, the *Z* number does not depend on the fluid velocity (*U*), therefore *Z* only accounts for the physical properties of the fluid and the characteristic length.^89^
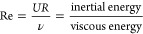
1
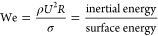
2
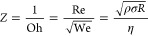
3Ink droplets are considered printable in a DOD inkjet printer when *Z* is within the range of 1–10.^153^Figure 5 illustrates the classified regions for printability and drop formation on the We versus Re chart. In the high-viscous region (*Z* > 1), the fluid cannot form droplets, while in the low-viscous region (*Z* > 10), inkjet printing would result in the formation of satellite droplets.^154^ However, it is important to note that the parameter Oh^–1^ provides only an approximate quantification of ink printability and other factors may also influence the printing process.

When a droplet falls onto a surface at a speed exceeding a critical limit or when We^1/2^Re^1/4^ > 50, splashing occurs. Additionally, a minimum Weber number value of 4 is needed for enough kinetic energy in the ink flow to overcome surface tension, leading to the formation of smaller diameter droplets.^154,155^ These threshold values define regions where drop formation and DOD inkjet printing can be achieved, as shown in Figure 5. Moreover, they can serve as predictors of the printability of newly formulated inks. For further exploration, comprehensive textbooks and reviews are available to understand the fundamentals of inkjet ink preparation, which can be valuable resources for future studies.^153−155^

Furthermore, selection of a type of solvent is based not only on the properties of the solvent to create a stable ink but also on its chemical compatibility with the print head used for printing, as not all commercial print heads are compatible with every type of solvent. For research applications, the Dimatix Samba materials cartridge is commonly used because of its small ink reservoir, low price, and aqueous compatibility. For commercial printing a wide range of different print heads are available, with both aqueous and nonaqueous compatibility.^156^

An overview of common solvents that have been used for inkjet-printed MOFs is provided in Table 2. Aqueous (water-based) and nonaqueous solvents, as well as their mixtures, are commonly used in MOF inks. Aqueous inks generally exhibit lower viscosity and evaporation rates, along with higher surface tension compared to nonaqueous solvents.^157^ However, the properties of aqueous inks can be adjusted to meet specific requirements of inkjet printers by incorporating cosolvents such as alcohols, glycols, and surfactants.^24^ Nonetheless, it is important to note that water-based inks tend to have slower drying rates on nonporous substrates like glass.^25,64^ In works reported by Xu et al.,^96^ Liang et al.,^81^ and Lv et al.,^77^ water was chosen to be the only solvent for developing MOF inks. Water is a solvent with a low viscosity (0.895 mPa·s at 25 °C) and high surface tension (71.97 mN/m at 25 °C). When assuming using a normal nozzle with a diameter of 20 μm, water-based ink either cannot be printed at all or satellite droplets will form depending on the fluid ejection velocity. In all cases, neither the viscosity, surface tension, and nozzle size nor *Z* values of the MOF inks were provided or discussed. However, it could be speculated that the high concentration of MOFs (mg/mL) increases the viscosity of the MOF inks, thus compensating the inks into the printable range. According to the Einstein equation (eq 4), the viscosity of a suspension (η) is affected by the viscosity of the base solvent (η_0_) and the volume diffraction of the added particles (ϕ), although this equation might be too idealized.
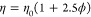
4

**Table 2:** Common Solvents and Selected Properties^163^

Solvents	Density (g/mL)	Boiling Point (25 °C)	Evaporation Rate (BuAc = 1.0)	Surface tension (mN/m at 25 °C)	Viscosity (mPa·s at 25 °C)
Water	1	100	0.36	71.97	0.895
Ethanol	0.789	78.2	1.7	21.97	1.074
Ethylene glycol	1.114	197.3	N.A.	47.99	16.1
Diethylene glycol	1.118	245.8	N.A.	48.5	30.2
Glycerol	1.261	290	N.A.	76.2	934
Isopropanol	0.785	82.3	1.6	20.93	2.038
*N*,*N*-Dimethylformamide	0.945	152.8	0.03	36.42	0.802
Dimethyl sulfoxide	1.1	189	0.026	42.9	1.991
Acetonitrile	0.783	81.6	5.79	29.04	0.35
Benzonitrile	1.01	190.7	0.13	38.79	1.001

**Figure 6:**
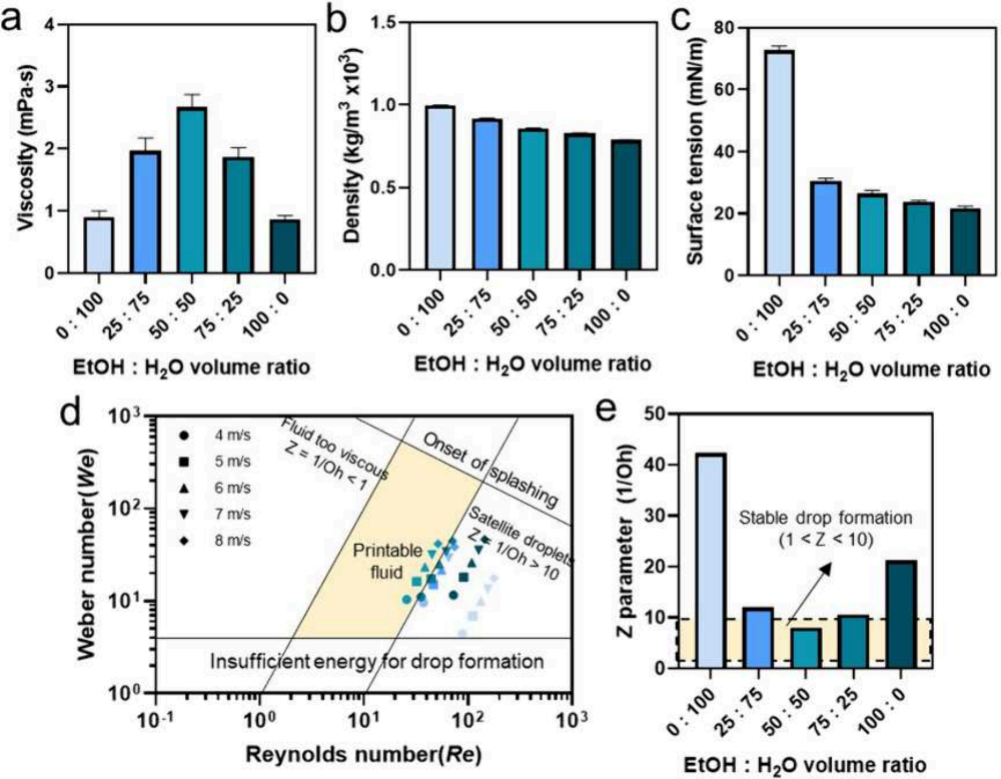
Properties of a series of H_3_BTC ethanol/DI water inks. Comparison of (a) viscosity, (b) density, and (c) surface tension, (d) ink printability map, and (e) calculated *Z* parameter. Reproduced from ref (98). Copyright 2024 American Chemical Society.

Organic solvents, including alcohols, glycols, and DMF, are also commonly utilized as carrier fluids for MOF inks. These solvents are carefully chosen to enhance ink stability and ink jetability, increase the viscosity of ink, and reduce the surface tension of solvents compared to pure water-based ink. Most organic solvents have higher viscosity and lower surface tension compared with water, affecting the Reynolds number and Weber number, respectively. The map of the common organic solvents and water used for developing MOF/COF inks is plotted in Figure 5 (assuming the nozzle is 20 μm and fluid speed is 10 m/s). As can be seen from Figure 5, most organic solvents have a lower Reynolds number (Re) than water except DMF. As the *Z* value is inversely proportional to the Re number, employing organic solvents into the inks can therefore decrease *Z* values compared to pure water. Besides the Reynolds number, the Weber number (We) is also affecting the *Z* values. The Weber number is dominated by the density and surface tension of solvents (besides the variables caused by inkjet printer). As shown in Figure 5, the organic solvents also have a higher Weber number compared with water, which can also lead to a lower *Z* value. Thus, in most cases, a combination of different solvents was used to achieve printable inks. Although the *Z* value determines the printability, only a few recent framework materials reported the *Z* value.^61,98^ In the work by Kim et al.,^98^ deionized water and ethanol with various ratios were combined to develop a 1,3,5-benzenetricarboxylic acid (H_3_BTC) ink. It is found that when the volume ratio of ethanol/DI water is 50:50 (v/v), the organic linker ink is located in the printable zone with different ejection speeds from 4 to 8 m/s (see Figure 6), but for inks mixed with other ratios, *Z* values are not located in the printable range. In the work reported by Teo et al.,^61^ two inks were developed using glacial acetic acid as solvent. It is found that the *Z* values of the developed inks are about 40, which is outside the printing region, yet successful printing was still achieved by adjusting the actuation voltages.

It can be noticed from this map that several solvents (e.g., isopropanol (IPA), ethanol (EtOH), dimethyl sulfoxide (DMSO), and glycols) are already printable or at the periphery of the printable region. However, another factor, namely, evaporation rate, needs to be taken into consideration when choosing solvents (either individually or in combination). Slower-drying solvents (glycols, DMF, DMSO, etc.) may prolong drying time and require additional energy for evaporation, while volatile solvents facilitate quicker solvent removal and thus enable efficient deposition of multiple layers. However, it is important to prevent rapid evaporation of the solvent (e.g., low-molecular-weight alcohols) at the nozzle, as it can result in clogging or nonuniform printing layers.^60,102^ Mixing solvents with low and high evaporation rates can help to minimize the negative aspects of each. For example, acetonitrile has a high evaporation rate, resulting in rough films. Addition of benzonitrile, which has a lower evaporation rate, resulted in smoother films.^60^ The problems that might be caused by less or high evaporation rates have often not been explicitly discussed in any MOF/COF inkjet printing papers so far, but they are certainly important factors. Moreover, the interaction between the chosen solvents and the substrate significantly impacts adhesion and printability on the surface.^88,158^ Therefore, solvent selection not only influences the feasibility of printing but also affects the quality and uniformity of the deposited layers on substrates, ultimately leading to improved print outcomes.^76^

The choice of solvent and additives in the ink formulation can have a significant impact on the stability and integrity of the MOF/COF material. In the case of printing MOFs/COFs with polar solvents such as water, some MOFs/COFs may undergo partial degradation; examples are HKUST-1 or boronate ester COFs with water as solvent. This degradation can result in the loss of the original surface area and potentially affect the performance of the MOF/COF in the printed film.^159^ Hence, solvents in which the MOFs/COFs remain structurally intact should be used to ensure optimal printing outcomes.

For direct printing, suspension of MOF particles in common solvents can be quite challenging, particularly in the context of preparing inks for inkjet printing.^160,161^ One important factor to optimize is the MOF concentration in the ink formulation, as high concentrations can lead to increased sedimentation and viscosity due to particle–particle interactions, which can thus lead to clogging of the printer nozzle.^162^ Colloidal stability of the ink is thus vital to ensure a consistent printing performance. Note Section 3.2.1.2 for strategies on how to achieve colloidally stable MOF and COF solutions.

In the context of reactive printing, a combination of solvents has been shown to be successful in maintaining the stability of certain ink formulations. For instance, the inclusion of ethylene glycol (EG) has been shown to significantly enhance the stability of the HKUST-1 precursor solution. In this case, the precursor solution, composed of copper salt and ligand in DMSO, EtOH, and EG, remained stable without any signs of precipitation even after extended storage periods up to 8 months under ambient conditions.^39^ Similarly, for the reactive inkjet printing of ZIF-8 precursors, a combination of DMSO, EtOH, and EG in specific volume ratios (4:9:6) was found to be the optimal solution, ensuring successful printing and the formation of desired ZIF-8 particles.^97^

In reactive inkjet printing, it is crucial to carefully balance the ratio of metal to ligand to achieve a high yield in MOF film production^97^ or the ratio between linkers for COF film production. Additionally, the solvents selected for precursors should be suitable for MOF/COF growth after printing, especially in the case where the precursors are printed separately. However, if the precursors are premixed before printing, there might be a risk of blocking nozzles if the crystallization kinetics are fast. This means that one should consider the solubility and deprotonation rate of the ligands in solvents as both factors play a crucial role in nucleation and crystallization of MOFs. This might involve the use of polar solvents such as water, alcohol, or DMF; however, choices vary from MOF to MOF. As imine-based COF formation is acid catalyzed, acetic acid as a (co)solvent is important to facilitate good film formation.^61^ All in all, the discussion above underscores the importance of optimizing ink formulations for successful reactive printing of MOFs/COFs. Different strategies regarding selecting solvents can be adopted for reactive printing and direct printing though.

#### 3.2.3 Additives

Ink formulations might require the addition of additives such as surfactants, copolymers, dispersing agents, and acids/bases in small amounts to enhance ink stability and prevent particle agglomeration. These additives play a crucial role in modifying the viscosity and surface tension of the ink, which are essential for achieving smooth dynamics of microfluidic droplets and high-resolution printing.^89,94,97^ For example, sodium dodecyl sulfate (SDS), an anionic surfactant, was added to the lanthanide coordination polymers mixed solution containing ethanol, glycol, glycerin, and diethylene glycol to adjust its viscosity and surface tension.^94^

The addition of suspension agents is often necessary to maintain the stability of the ink formulation. An alternative method to prevent particle aggregation in ink formulations involves controlling particle sizes and ensuring suitable surface charge properties.^78^ This is commonly accomplished by introducing an acidic medium, such as HCl or HNO_3_, which induces charges on the particle surfaces, leading to electrostatic repulsion forces between them. These repulsion forces effectively hinder particle clustering during the printing process. However, this approach has not been used in any of the MOF or COF papers. Nevertheless, it is crucial to be mindful that the inclusion of acidic additives in the ink formulation may entail potential drawbacks, including the potential risk of structural degradation or the loss of functionality in the MOF material^164^ or causing damage to the nozzles.

### 3.3 Substrate

We will first discuss the general theoretical considerations that are relevant to the choice of substrate and its interaction with the ink before discussing specific examples of MOF/COF inks used on various substrates.

Substrates utilized for different applications can be porous or nonporous, rough or smooth, flexible or rigid.^165^ To achieve uniform and defect-free printed layers, it is essential to thoroughly clean the substrates before surface treatment and printing. This can be done through rinsing with water/alcohol and utilizing sonication.^52,75,79^ By ensuring a clean substrate surface, we can improve the effectiveness of subsequent surface treatments and the quality of the printed layers can be improved.

Wetting refers to the ability of a liquid (ink) to maintain contact with a solid substrate due to intermolecular interactions between the liquid and solid interface. When ink droplets come into contact with the substrate surface, they undergo a dynamic spreading process. Initially, the droplets spread on the substrate, driven by their kinetic energy, known as inertial spreading. This is followed by capillary spreading and/or recoil, where the wetting of the ink on the substrate surface determines the extent of spreading. Finally, the ink droplets reached a state of thermodynamic equilibrium with the substrate and the surrounding environment. These stages of droplet spreading are complex and are influenced by various factors such as the ink properties (surface tension, viscosity), substrate properties (surface energy, roughness), and environmental conditions (temperature, humidity).^166^

Understanding and controlling the equilibrium phase of droplets on the substrate is critical for achieving desired print quality and ensuring proper layer adhesion.^167^ The equilibrium state of a droplet on the substrate (wettability) can be characterized by the contact angle at the liquid–gas–solid interface. A high contact angle (i.e., >70°) implies poor wetting, which leads to reduced adhesion and irregularities in the printed layer. Conversely, a low contact angle indicates good wetting, resulting in uniform ink spreading and strong substrate adhesion. However, if the contact angle is too low (i.e., <5–10°), the droplet may spread excessively over the surface, reducing spatial resolution and resulting in overly thin layers.^168,169^ By optimizing the wetting behavior, one can improve the print quality, achieve uniform and defect-free printed layers, and enhance the overall performance of the inkjet printing processes.

In practice, surface wettability can be enhanced by modifying either the solid–gas interfacial energy of the substrate surface (e.g., surface energy and roughness) or the liquid–gas interfacial energy of the ink formulation (e.g., surface tension, viscosity). The wetting characteristics of the substrate play a crucial role in the deposition of ink droplets during printing.^1^ Good wetting typically occurs when the surface tension of the ink is lower than that of the substrate. For instance, printing aqueous-based inks (with a surface tension of ∼72 mN/m at 25 °C) on untreated polymeric substrates (with low surface energies of 20–50 mN/m) may result in poor wetting.^170^ To improve wetting in such cases, nonaqueous-based inks with lower surface tensions, such as alcohols, can be used. Alcohols can also be used as surfactants in aqueous inks to lower the surface tension.

In the cases where the droplets form a pinned contact line on the substrates, a ring-stain deposit might form and it is usually referred to as a “coffee ring”.^171^ The coffee-ring effect (CRE) in inkjet printing is an undesired phenomenon where solute particles accumulate primarily at the droplet contact line, forming a ring-like stain. CRE is driven by capillary flow, which arises from a differential evaporation rate across the droplet, directing particles toward the pinned contact line.^172^ The evaporation rate of the solvent at the periphery is higher than for other regions of the droplet, resulting in complex flow patterns inside the droplet including capillary flows toward the contact line. These capillary flows can transport smaller particles toward the contact line, where they will be deposited in either an ordered (hexagonal or face-centered) format or a fully disordered format, depending on the flow rate.^173^ The formation of the coffee-ring effect is also influenced by several factors beyond capillary flow, including contact line pinning, contact angle hysteresis (CAH), thermal Marangoni flows (induced by surface tension gradients), and interactions between particles and the substrate (electric double layer).^172^ Various strategies can be implemented to suppress the CRE and achieve uniform thin films. The first approach is to prevent contact line pinning, which can be achieved by minimizing CAH using low-CAH superhydrophobic surfaces or through CAH suppression using electrowetting. The second strategy involves disrupting capillary flow to reduce solute transport to the droplet edge; this can be achieved by inducing internal flow fields within the droplet through mechanisms like Marangoni flows, electroosmosis, electrowetting, or acoustic streaming. The third strategy involves manipulating particle interactions within the droplet to prevent their transport to the contact line, such as promoting particle aggregation at the solid–liquid interface or modifying capillary flows through particle–liquid interactions. These techniques, combined with theoretical models and experimental approaches, can effectively reduce the coffee-ring effect, leading to more uniform film deposition for advanced MOF and COF applications. For further details, we refer readers to the review by Mampallil et al.^172^

Inkjet printing has proven to be a successful technique for depositing MOF materials onto a wide range of substrates, including types of paper,^39,47,79−81,93,94,^ polymeric substrates (plastic, film, foil, and membrane),^39,47,79,80,97^ glass,^64,78^ inorganic substrates,^75,77,95^ textiles^39^ and COFs on acetate paper,^53^ SiO_2_,^53^ and glass.^61^ However, it is important to note that most research has been concentrated on flexible porous substrates, where good wettability and adhesion are more easily achieved than those on smooth substrates. For example, when substrates were paper- or porous polymer-based, distinctly different MOF/COF ink recipes have been developed, as there are fewer concerns on the substrates’ side. Reactive inkjet printing of ligands for RT-COF-1 was achieved by depositing a stoichiometric DMSO solution of 1,3,5-tris(4-aminophenyl)benzene (TAPB) and 1,3,5-benzenetricarbaldehyde (BTCA) using a commercial inkjet printer on both rigid SiO_2_ surfaces and flexible acetate paper. On both substrates, the printed patterns exhibited significant uniformity. However, higher resolution was obtained on the flexible acetate paper, with dot arrays measuring 40 μm in diameter, compared to the 70 μm in diameter dot arrays of RT-COF-1 produced on the SiO_2_ surface. In the case of inkjet printing boronic-acid-rich metal–organic frameworks (BMOFs), water was used to develop MOF inks. Successful deposition of BMOFs onto filter paper demonstrated good adhesion and homogeneous dispersion. However, the BMOFs tended to aggregate when printed on a poly(vinylidene fluoride) (PVDF) membrane. As mentioned previously, water has a higher surface tension than PVDF films, which combined with the hydrophobic nature of PVDF, would lead to poor wetting. The choice of aqueous ink and PVDF substrate in combination is therefore inadvisable.^81^

**Figure 7:**
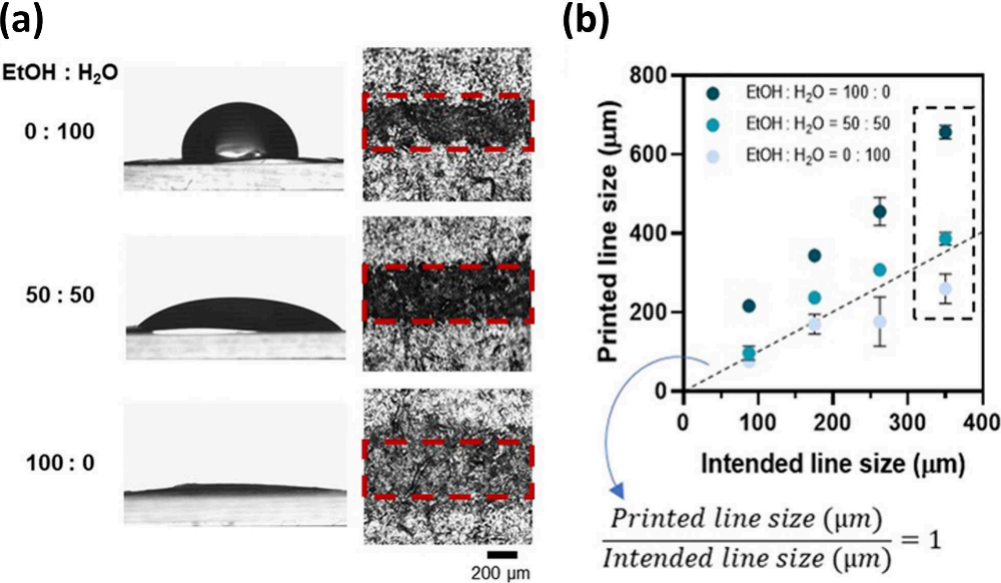
(a) Contact angle and wetting regions and of EtOH/DI water-based ink on the Cu^2+^-exchanged TCNF film, (b) Comparison of the difference between printed line size and intended line size of ink. Reproduced from ref (98). Copyright 2024 American Chemical Society.

In contrast, a good wettability can also cause problems. In another study by Gregory and co-workers,^64^ DMF was utilized to develop precursor inks to deposit MOFs on glass with reactive inkjet printing. Although the authors did not state this explicitly, it can be surmised that in such an ink–substrate combination, the inks would have a low contact angle on glass substrates, leading to a low spatial resolution of the printing. This can be observed in several patterns printed in their work. This again emphasizes the importance of studying the wettability of inks on various substrates to ensure a good printing fidelity. However, only recently, one paper measured and reported the contact angle of the inks.^98^ In this work, three different ligand (H_3_BTC) inks consisting of ethanol and water (0:100, 50:50, and 100:0) were dropped onto a Cu^2+^-exchanged TCNF film. It was observed that as the ratio of water increased, the contact angle increased (wetting decreased) and vice versa (Figure 7). The wettability of the ink on the Cu^2+^-exchanged TCNF film surface significantly influenced the spreading of the ink, which ultimately affected the size of the printed features. Higher wettability led to larger line features during printing.

In terms of adhesion, a notable example of adhesion is the printing of small MOF-525 crystals on indium tin oxide (ITO).^78^ The small MOF-525 crystals have a tendency to stack closely together, which resulted in the formation of a compact thin film with a larger contact area with the substrate. This intimate contact and increased contact area contribute to improved adhesion between the MOF film and the substrate, ultimately influencing the overall mechanical properties of the thin film structure.

Note also that the substrate’s surface energy can be enhanced through surface treatment techniques like physical treatments or chemical modification.^174^ However, up to now, these surface modification techniques have not been used when printing MOFs or COFs. These substrate modifications can activate the substrate’s surface and enhance the bonding between the substrate and the functional groups present in the ink solution. A common practice to improve ink adhesion and wetting properties is via surface modification of the substrates before printing. For example, various techniques such as plasma treatment, UV/ozone-curable coatings, corona discharge, and flame treatment can be utilized to activate the substrate to make it more receptive to the ink and promote better adhesion.^24,52,61,98,168^ Chemical functionalization is another approach that can be used to enhance wetting properties and increase the affinity between printed materials and substrates.^23^ Adhesion promoters, such as silane-based chemicals, can be employed to functionalize dispersed particles or the substrate itself. This enhances the interaction between the ink and the substrate, improving wetting and adhesion.^175,176^ The choice of adhesion promoter depends on the specific ink and substrate materials being used. In summary, the approaches mentioned above present an opportunity for the MOF/COF field to be explored in inkjet printing.

## 4 Printed MOF and COF Properties

To ensure the effective utilization of printed MOF/COF layers in various applications, specific properties need to be carefully considered such as porosity, mechanical strength, thickness, uniformity of the film, and patterning.

### 4.1 Porosity

MOFs and COFs are renowned for their high porosity, which provides a large surface area for catalytic reactions and efficient diffusion and adsorption of guest molecules.^2,177^ However, there is limited literature that investigates the porosity of printed MOF or COF films^61,64^ as the very small amount of material in sub-micrometer thick films excludes the use of most bulk techniques to assess the porosity. For example, the widely used techniques of N_2_ physisorption cannot be reliably used on low amounts of total pore volume of printed MOF thin layer on the substrate.^178^

Several physisorption- and spectroscopic-based techniques have been adopted to access the porosimetry of MOF films. Physisorption approaches are based on employing various probes and measuring the adsorbed quantity of a probe molecule through manometric/volumetric (e.g., Kr physisorption, KrP), gravimetric (e.g., quartz crystal microbalance, QCM), or spectroscopic (e.g., ellipsometry, EP) methods. These methods are performed as a function of the adsorptive relative pressure at a constant temperature to generate adsorption/desorption isotherms. From these isotherms, metrics like pore volume and specific surface area can be derived by using appropriate models. However, limitations arise in evaluating pore size in thin films due to restricted instrumental sensitivity and the need for advanced methods in data analysis.^179^ Although the pore size cannot be determined directly via the above techniques, positron lifetime annihilation spectroscopy (PALS) is able to provide this information. In PALS, this circumvents the need for molecular probes by bombarding samples with positrons to determine pore sizes. While PALS has shown promise in evaluating porosity in crystalline and glassy MOF powders, its potential for MOF thin films remains largely unexplored, necessitating further investigation and comparison with adsorption-based methods.^179^

The four methods (KrP, QCM, EP, and PALS) can provide complementary information on porosimetry of thin films, yet each of them has its own pros and cons. In terms of techniques for assessing porosimetry, there is not one specific technique that is superior to the others. They can offer complementary information on the MOF film. However, the availability of the four techniques follows the order QCM > KrP > EP ≫ PALS. However, do notice that when assessing porosity with the QCM technique, the MOF/COF film will be deposited on QCM crystals rather than the substrates of applications, and the film quality might vary on different substrates. For further details about the techniques to characterize the porosimetry of MOF/COF thin films, the work of Stassin et al. is suggested.^179^

Only one recent work studied the porosity of printed MOF layers using N_2_ adsorption measurements.^98^ The authors did not disclose the specific amount of MOFs printed, but typically, the amount required for accurate N_2_ adsorption measurements is in the range of hundreds of milligrams, which is a considerable amount to be obtained by inkjet printing. Besides this case, only a few studies studied the porosity in MOF layers through indirect methods, such as selectively encapsulating dyes of different sizes based on the aperture sizes of a Cu_3_(BTC)_2_ MOF^64^ or using the colorimetric and fluorescent detection of NH_3_ vapors for Mn-BDC and Tb-BTC films.^47^ For some applications, like anticounterfeiting,^76,79^ the porosity might not be a critical factor, yet for many other applications it will be; in addition it is also a parameter to assess the quality of the synthesized MOF/COF crystal structure. The porosity can be properly assessed and used only after solvent removal.

Post-treatments, including conventional activation or calcination, activation by solvent exchange, activation by freeze-drying, activation by the use of supercritical carbon dioxide (ScCO_2_), and activation by chemical treatment (acid treatment), are employed mostly after MOF/COF synthesis to enhance high surface area with porosity that is permanent. It is also essential to select or develop activation methods that do not bring about structural collapse of the framework, which leads to partial or total loss of porosity. The specific temperature and heating conditions depend on the solvent properties, the nature of the MOF, and the substrate used.^180−183^

After inkjet printing, it is crucial to effectively remove the solvent or precursors from the deposited MOF layer. The commonly used methods to get proper porosity for printed MOF/COF include drying in an oven^47,77,96,98^ or solvent exchange.^39^ For instance, the removal of entrapped solvents such as DMSO, EtOH, and EG from printed patterns of Co-MOF-71, Fe-MIL-101, Cr-MIL-101, and HKUST-1 was achieved by heating them at elevated temperatures (e.g., 60, 100, and 150 °C) for 30 min.^47^ These MOFs exhibited some color change at temperatures above 100 °C. Another example is the work by Xu et al.;^96^ after inkjet printing ZIF-8 onto a microcantilever, it was then dried in an oven at 60 °C to remove the solvents inside the pores. However, it is essential to carefully determine the temperature and duration of the heating process to ensure complete solvent removal without damaging the MOF layer or substrate.

**Figure 8:**
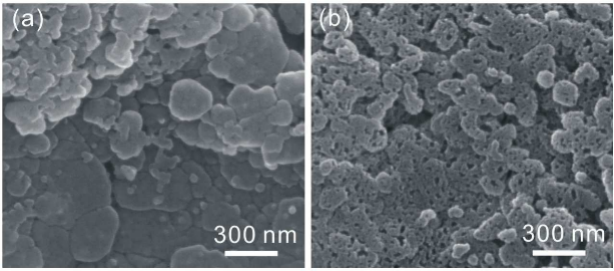
SEM images of as-printed HKUST-1 on foil (a) before and (b) after solvent exchange. Reproduced with permission from ref (39). Copyright 2013 Wiley VCH.

Solvent development, also called solvent exchange, might be needed regularly to remove the solvent after printing. When less volatile solvents are used, it might be necessary to immerse the printed film into a more volatile solvent for solvent exchange, after which a high-temperature or vacuum treatment can be used to remove the more volatile solvent. An example is the reactive printing of HKUST-1 from an ink including EG. The latter is removed via immersing the printed pattern in a methanol solution for 30 min. During this process, interestingly, mesopores were created (Figure 8), leading to hierarchical porosity in the printed HKUST-1.^39^

In some of the published literature on printed MOFs and COFs, additives are used. It is important to carefully consider whether these iodide ions may cause pore blockage. In inkjet printing of MOFs surfactants like sodium dodecyl sulfate and Triton-X-100 could be used, which provides an additional reason to assess the porosity. So far, binders have not been used in MOF/COF ink formulations, yet, for direct inkjet printing on smooth, nonporous substrates, one may expect that binders (e.g., polymers) are needed for long-term retainment of the MOF/COF crystallites on the substrate. Such binders will cause a challenge in terms of keeping the pore space of the framework materials accessible,^184^ again underscoring the importance of both carefully designing ink formulations and the assessment of the porosity after printing.

### 4.2 Patterning

**Figure 9:**
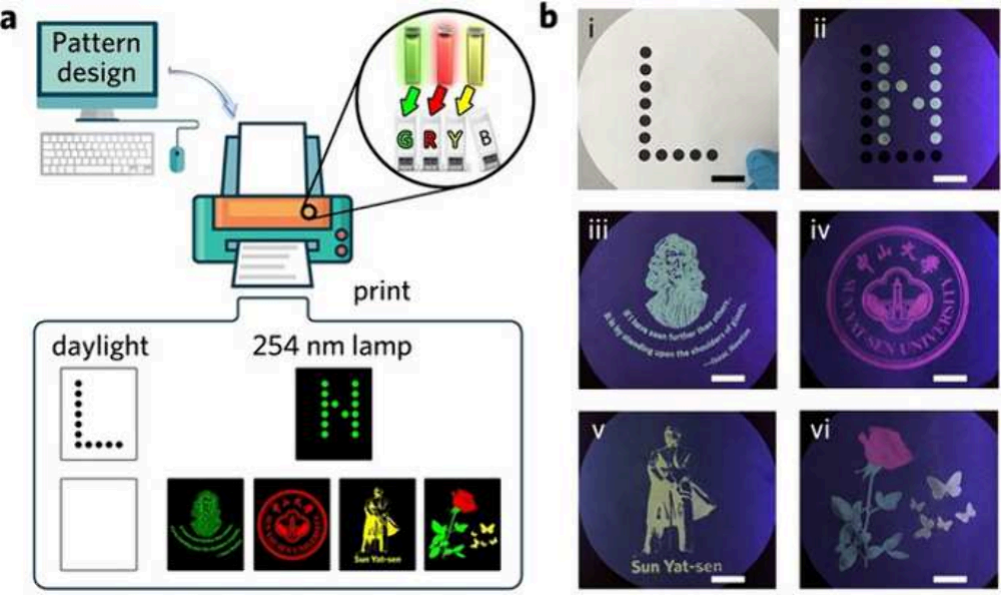
(a) Schematic illustration of pattern encryption with green, red and yellow security inks. (b) Photographs of printed MOFs patterns under daylight or 254 nm lamp irradiation. Scale bars: 2 cm. Reproduced with permission from ref (94). Copyright 2020 John Wiley & Sons.

**Figure 10:**
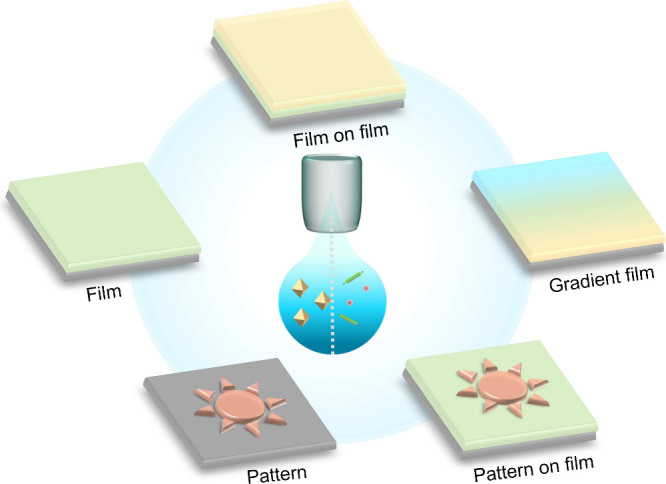
Prospects of MOF patterns/films using inkjet printing technique.

Beyond the controlled deposition of MOF thin films, or even stacked layers of films, a specific strength of inkjet printing is the capability to print patterns, where MOF films are selectively deposited in specific areas or patterns onto a substrate.^25,39,47,52,64,80,81,97^ So far, patterned MOFs have been reported specifically targeting applications as sensors^75^ and anticounterfeiting measures^76^ (Figure 9). For the latter, luminescent MOFs have been effectively incorporated into security ink using the precise inkjet printing technique to create specific patterns, such as text or barcodes, to enhance security features and enable authentication processes.^76,94^Figure 10 shows the potential of depositing MOFs and/or COFs using inkjet printing techniques for patterning and fabricating films. Inkjet printing of MOF/COF patterns facilitates the integration of MOF/COF films into complex device architectures or functional systems. Indeed, for integrated device fabrication, miniaturization in the fabrication of electronic, sensing, and microfluidic devices, with precise alignment and integration of multiple materials, is essential.^74,78^

Moreover, inkjet printing of MOF/COF patterns allows for scalability, as it enables the replication of patterns over large areas or many devices, making it possible to produce MOF-based devices and structures in large amounts (scalability). By depositing MOF material only in the desired pattern, excess material is also avoided, leading to cost savings and improved efficiency in material usage.^69−71^

It is also possible to create patterns where parts consist of different MOF or COF structures such that different areas of the film will exhibit different adsorption, catalytic, or sensing properties. It is even possible to let the composition or properties of the MOF film vary gradually across the pattern, enabling enhanced functionality or performance.^24,39,74^ Note that the inkjet process can create surface-anchored metal organic framework (SURMOF) gradients by simply defining a color gradient (e.g., from black to gray, as related to the amount of material deposited) for reactive inkjet printing of precursor of HKUST-1.^39^ It is also feasible to introduce gradual variations in the composition or properties of the MOF film within a pattern, enhancing functionality or performance through the combination of different ligands and metal ions while preserving the fundamental MOF structure. The principle of “isoreticular substitution” allows for the creation of diverse frameworks with varying functionalities. Additionally, the concept of “multivariate” frameworks (MTV-MOFs) has emerged, enabling the blending of different ligands or metal ions within a single framework while maintaining the same structure. This modular approach in reactive inkjet printing offers the possibility of using the same precursor inks to print different MOFs and employing spatial control to create gradients in transitioning from one structure to another. In a practical demonstration, six inks containing DMF solutions of Cu, Zn, and Co acetate salts, along with ligands (BDC, ABDC, BIM), were combined through reactive inkjet printing, producing well-known MOFs such as Cu(BDC), Cu(ABDC), Zn(BIM)_2_, and Co(BIM)_2_. Adjusting ink ratios facilitated the achievement of gradients in ligand composition, from 100% Cu(BDC) to 100% Cu(ABDC), or gradients of metal ions by variation of the ratio of Zn and Co inks added to the BIM ink. Although the gradient printing was realized by premixing components within the inks in these examples, an upgraded system with three nozzles could potentially refine the process further by altering the number of droplets for each ink containing only one reactant, providing enhanced control over the printing outcome.^64^

In another study, inkjet printing technology was employed to fabricate patterned electroactives on a continuous MOF film in an efficient and rapid procedure. The focus of the research was on attaching redox-responsive ferrocene carboxaldehyde (Fca) covalently to UiO-66-NH_2_ on FTO glass, creating a redox-active MOF surface with desired patterns. This approach allowed for precise control over the spatially postsynthetic patterning of MOF films, enabling the development of a redox-active MOF surface suitable for electronic applications. A resolution of a few hundred micron was reached with use of a nozzle with 35 μm.^25^ However, in another study, the printing resolution for inkjet printing H_3_BTC on a Cu^2+^-exchanged TCNF film was determined to be 83.8 ± 2.9 μm. This resolution was achieved using the smallest line width (1/4 points) and a 20 μm nozzle diameter of the inkjet printer.^98^

### 4.3 Mechanical, Thermal, and Washing Stability

The mechanical strength of the printed MOF layer is essential for its stability and durability during applications under operating conditions, such as temperature, pressure, and mechanical stress. The substrate and its adhesion to the MOF film play crucial roles in determining the mechanical properties of the film, especially considering the inherent fragility of thin films. As-prepared MOF layers generally exhibit limited adhesion to the substrate, which can impact the overall mechanical stability of the film.^76,79^ Measuring and quantifying the adhesion of films to substrates can be challenging, as there is currently no standardized quantitative approach available for comprehensive and comparative analysis and different tests may yield contradictory results.^185^ Consequently, there is a limited body of work on the stability of inkjet-printed MOF layers on substrates,^47,79,80^ warranting further exploration for various applications.

One commonly used technique to assess the adhesion properties of thin films is the scratch test. This test involves applying controlled mechanical force to the film’s surface and observing the response, such as the onset of cracking, delamination, or detachment.^89^ The scratch test provides valuable insights into the adhesion strength and integrity of the MOF film–substrate interface.^47,79,80^ While the scratch test is a conventional method, it is important to note that the interpretation of results should consider other factors, such as film thickness, substrate properties, and test conditions. The stability of printed patterns on substrates can be investigated using the tape scratching method, which has indeed been done in a few studies on inkjet-printed MOFs.^47,79,80^ In this method, a simple adhesive tape is applied onto the printed film and then gently scratched after a specific duration, for example 2 min. This adhesion-scratching process can be repeated multiple cycles. Afterward, the printed film is examined to assess the visibility of any damage caused by the scratching. For example, direct inkjet printing of Fe-MIL-101 on transparent PET and on opaque PET showed some visible damage after the fourth cycle, and the patterns remain roughly intact for practical usage even after the eighth cycle. The tape scratching method provides a simple and practical way to evaluate the adhesion and durability of the printed MOF patterns on the substrate.^47^

In one of these studies, focused on thin films of various MOFs, including Cr-MIL-101, Mn-BDC, Fe-MIL-101, Co-MOF-71, Ni-BDC, and HKUST-1 on PET substrates, also the thermal stability of printed MOF films was evaluated by subjecting them to elevated temperatures, typically ranging from 60 to 150 °C, for a specific duration, for example 30 min. During the heating process, color changes were observed in the MOF patterns when subjected to temperatures exceeding 100 °C. Notably, the colors of the Fe-MIL-101 (yellow), Co-MOF-71 (purple), Cr-MIL-101 (green), and HKUST-1 (blue) patterns transformed into dark brown, blue, dark green, and dark blue, respectively. It is mentioned that this alteration in color is attributed to the removal of entrapped solvents (DMSO, EtOH, EG) and water present in the initially moist printed patterns, leading to coordinatively unsaturated metal sites. It is worth noting that certain MOF materials might undergo changes in structural integrity, such as cracks or gaps in the film, during this process, potentially affecting their properties, yet an assessment of these was not included in the work.^47^

Goel et al.^47^ also tested the washing speed of these printed MOF films, which can be evaluated to assess their stability and adherence to the substrate. The films were immersed in DI water or ethanol for 2 min in Petri dishes for several cycles. Remarkably, no visible damage or peeling off of the patterns was observed even after 10 washing cycles, indicating a significant level of adherence stability of the MOF thin films on the PET substrates.^47^

In reactive inkjet printing, heat treatment is often necessary to facilitate the MOF crystallization. However, such treatment can cause thermal deformation and degradation of the substrate, making it essential to assess the substrate’s thermal stability. For direct inkjet printing of HKUST-1, rapid nucleation during the 80 °C thermal treatment resulted in densely grown, nonoriented crystals after multiple printing cycles, making individual crystals hard to distinguish. To counteract this, a slow nucleation procedure was implemented. The printed substrate was placed in a desiccator containing methanol vapor and left for 15 min after each printing step. This slower nucleation process led to the formation of (truncated) octahedral crystals after three cycles.^39^ Inkjet-printed COFs have not been tested for their stability so far.

### 4.4 Thickness and Uniformity of the Film

Achieving a controlled and uniform film thickness is essential for the consistent and predictable behavior of the printed MOF or COF material.^24,78^ The controlled MOF film thickness on surfaces allows for the spatial and morphological customization required for smart membranes, catalytic coatings, and sensing devices. Moreover, uniform and continuous and thus complete surface coverage enables enhanced performance in terms of gas storage, catalytic activity, and sensing capabilities.^39^

To ensure uniform film thickness, factors such as ink formulation, printing parameters, and substrate surface properties need to be optimized (Section 3). Controlling the ink concentration, viscosity, droplet size, and printing speed can contribute to a more uniform deposition of the MOF ink. Proper substrate preparation, such as surface cleaning and treatment, can also enhance the uniformity of the printed film.^24^ Notably, existing research often fails to report the effects of these parameters in the inkjet printing of MOFs or COFs, emphasizing the achieved uniform thickness with optimized parameters. This represents a missed opportunity to elucidate the impact of these critical factors, potentially serving as a valuable guide for future researchers. It is worth noting that the investigation into the effects of multiple printing cycles has been a primary focus in the existing literature.

**Figure 11:**
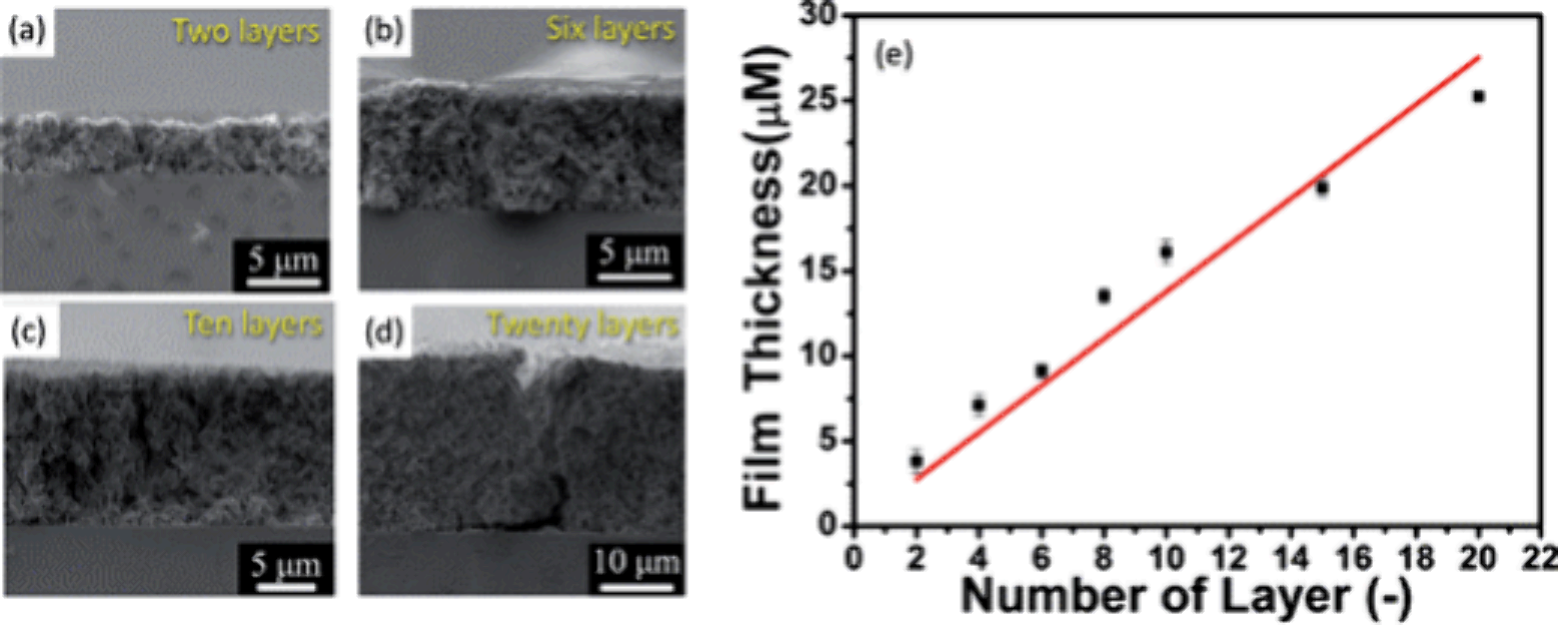
Cross-sectional SEM images of the inkjet printed M1.35 thin films with (a) two layers, (b) six layers, (c) ten layers, and (d) 20 layers. (e) Plot of film thickness estimated from SEM images vs number of printed layers. Reproduced with permission from ref (78). Copyright 2016 Royal Society of Chemistry.

To achieve the desired thickness of MOF thin films, repetition of printing and drying cycles is often employed. After the initial printing and drying cycle, the substrate is reloaded into the printer cassette, ensuring that it is positioned in the same location as in the previous cycle. This allows for the precise layering and accumulation of MOF ink to build up the desired film thickness.^39,47,80,98^ By increasing the number of cycles, the film thickness can be increased. It has, e.g., been observed that the thickness of the printed films exhibited a linear relationship with the number of layers in printing zirconium-based porphyrinic MOF (MOF-525) thin films (Figure 11).^78^ A similar linear relationship between film thickness and number of layers was found for RT-COF-1.^61^ However, the amount of HKUST-1 synthesized on Cu^2+^ exchanged TCNF film increased on the first, second, and fourth passes but stopped increasing after the fourth printing due to the amount of available Cu^2+^ in the Cu^2+^ exchanged TCNF film.^98^

In a study by Babal and co-workers, the coverage and uniformity of the MOF film were evaluated by comparing samples with different numbers of layers. For instance, a 3-layer sample of ZIF-70 was observed to cover the interdigitated comb-like electrode (IDE) sensor surface completely and uniformly. In contrast, a 1-layer sample exhibited incomplete coverage and uneven distribution. This observation suggests that multiple printing cycles and layering can lead to improved coverage and uniformity of the MOF film, ensuring a more reliable and effective sensor surface.^75^

In a different study by Goel et al.,^47^ the influence of the thickness of printed Mn-BDC and Tb-BTC patterns on the response time to NH_3_ gas was investigated. The study reports that both thicker (4 printing layers with MOF loading of 179 μg) and thinner (single printing layer with MOF loading of ∼45 μg) MOF films exhibited similar response times to NH_3_ gas within the measured range. Thicker films allowed for a higher MOF loading, however, leading to a more intense color generation or larger photoluminescence (PL) intensity change. The results highlight the importance of optimizing the film thickness for specific gas sensing applications to achieve the desired sensitivity and dynamic range.

**Figure 12:**
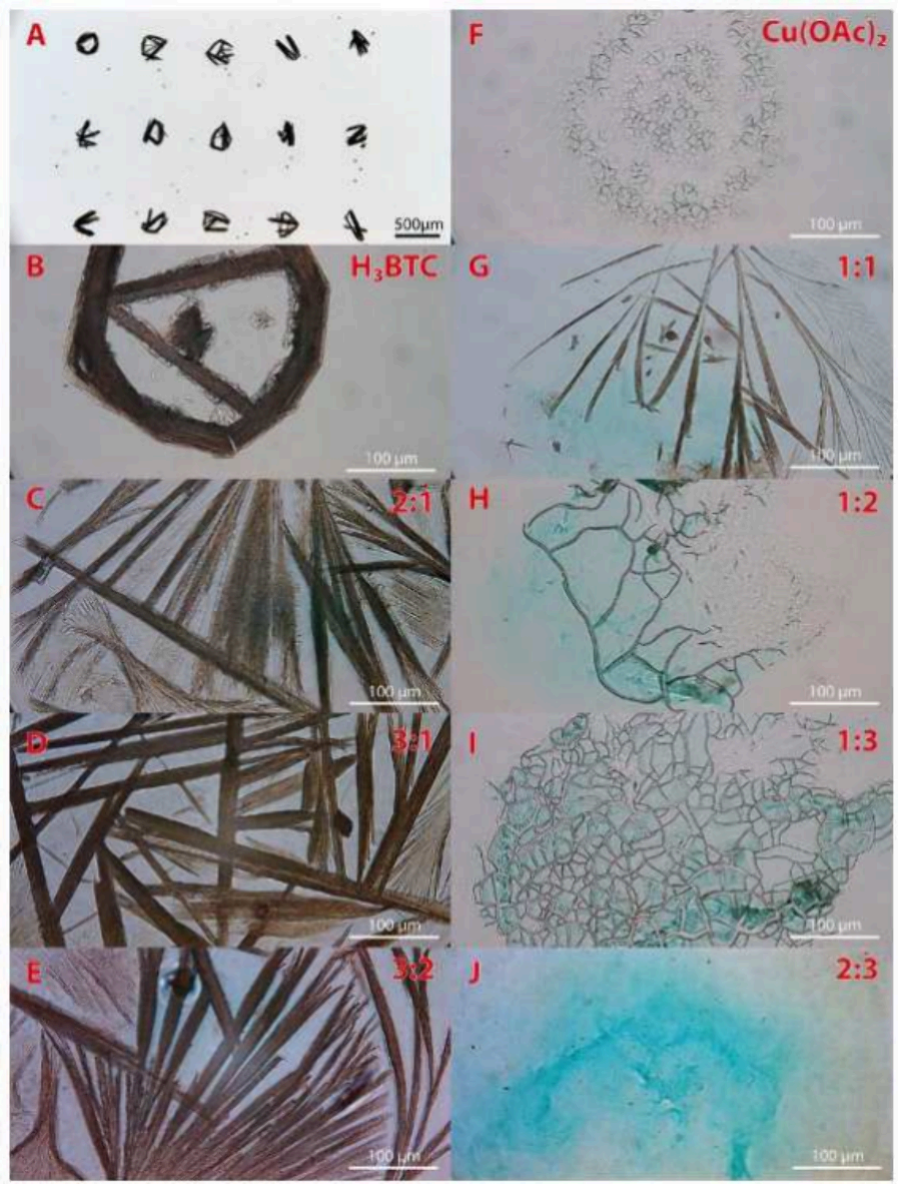
Microscope images of a dot array: (A) overview printed dot array on cover glass, zoomed-in image of printed (B) H_3_BTC 0.25 M in DMF and (F) Cu(OAc)_2_ 0.25 M in DMF, and different ligand to metal ratios printed, respectively, (C) 2:1, (D) 3:1, (E) 3:2, (G) 1:1, (H) 1:2, (I) 1:3, (J) 2:3. Reproduced from ref (64). Available under a CC-BY 4.0 license. Copyright 2023 by the authors. *Advanced Materials Interfaces* published by Wiley-VCH GmbH.

Particularly, in reactive inkjet printing, the metal node-to-ligand ratio significantly influences the uniformity of the MOF layer printing (Figure 12). In the case of reactive inkjet printing using H_3_BTC and Cu(OAc)_2_ at various ratios (1:1, 1:2, 1:3, 2:3, 3:2, 3:1, and 2:1), with the stoichiometry of Cu_3_(BTC)_2_ being 2:3, yielded blue printed lines consisting of microcrystalline material, closely resembling the color of bulk Cu_3_(BTC)_2_. Samples with an excess of metal ions (1:2, 1:3) did show this blue hue as well but exhibited more pronounced cracking upon drying. Conversely, when an excess of the H_3_BTC ligand was present (1:1, 2:1, 3:1, and 3:2), the formation of large needle-like crystals occurred, resembling those obtained by printing H_3_BTC alone. This phenomenon was attributed to the comparatively lower solubility of H_3_BTC in DMF compared to Cu(OAc)_2_, leading to the nucleation of H_3_BTC crystals, especially when present in excess.^64^ Careful optimization of the printing cycle is necessary to avoid issues such as cracking and to achieve the desired film quality and properties. For example, a study focused on printing MOF-525 demonstrated that a uniform thin film with a smooth surface could be deposited on ITO glass after printing 2 or 6 cycles. However, when the printing cycle was further increased to 10 layers, small cracks started to appear. Notably, serious cracking patterns were observed when printing 20 layers of MOF-525 thin film.^78^

### 4.5 Functionalization of Printed MOF/COF Films

**Figure 13:**
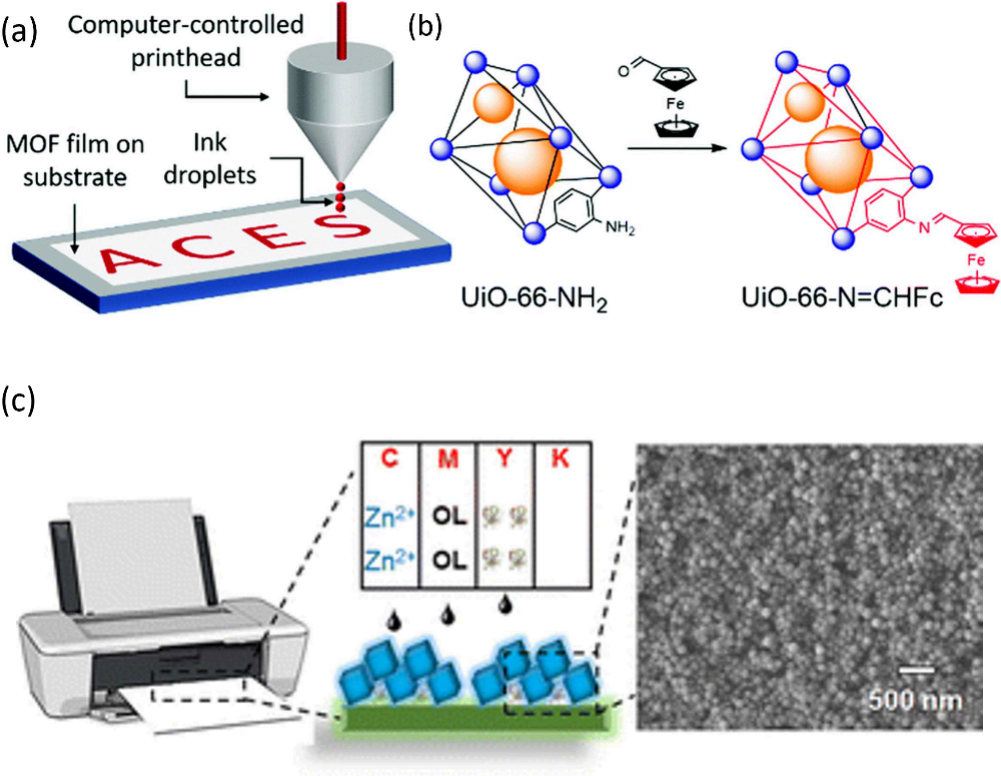
(a) Schematic of reactive printing postsynthetic modification (PSM) of MOF films. (b) Specific depiction of the PSM of UiO-66-NH_2_ using Fca. The small blue spheres represent the framework nodes, while the lines in the UiO-66-type structures represent functionalized linkers. For clarity, only one linker is shown. The size of the orange spheres corresponds to the larger octahedral and smaller tetrahedral pores present in UiO-66-type structures. Reproduced from ref (25) with permission from the Royal Society of Chemistry. (c) Reactive printing of ink solutions of ZIF-8 precursors zinc ions (Zn^2+^), 2-MeIM, and enzyme molecules on filter paper, OL as an abbreviation of the organic ligand 2-MeIM. Reproduced from ref (97). Available under a CC-BY 4.0 license. Copyright 2017 by the authors. *Bioresources and Bioprocessing* by Springer Nature.

Inkjet printing has also been employed for postsynthetic modification (PSM) of MOF films by printing a solution of functional materials directly onto the MOF film. This approach allows for the precise deposition of functional materials onto specific regions of the MOF layer, enabling the incorporation of desired properties or functionalities.^25,52^ An example of this includes the previously discussed (Section 4.2) rapid production of patterned electroactive MOF films using inkjet printing. In this case, redox-responsive Fca was covalently attached to UiO-66-NH_2_ on FTO glass through inkjet printing, resulting in the formation of patterned electroactive MOF films. Figure 13a,b illustrates the patterned electroactive MOF films achieved through inkjet printing.^25^

Another printing approach involves simultaneous printing and synthesis of the MOF layer with functional materials. By using separate precursor and functional material solutions, both are printed together at the same location, allowing for the *in-situ* synthesis of the MOF crystallites within the functional material. This strategy enables customization of the properties and functionalities of the MOF layer through inkjet printing, thereby expanding its potential applications in various fields. For instance, inkjet printing has been used to directly synthesize patterned enzyme-MOF composites on diverse substrates including paper and polymeric films. This was achieved by using inkjet-printed bioinks loaded with metal ions, organic ligands, and protein molecules, each loaded in different cartridges (Figure 13c).^97^

Functionalization of COFs via inkjet printing has not yet been reported. However, the colloidal growth of COFs with encapsulation of nanoparticles^113,114^ (further discussed in Section 3.2.1) does offer possibilities for reactive inkjet printing of COF encapsulated nanoparticles.

## 5 Summary and Perspective

In summary, inkjet printing is a promising technique for precise positioning and structuring of MOF/COF films, offering scalability and compatibility with a wide range of substrates, including flexible and porous materials. The direct-ink approach allows for printing of presynthesized MOFs and COFs dispersed in an ink solution, while reactive printing involves printing of precursor solutions and subsequent curing to synthesize the MOF or COF on the substrate. For the former type, control of the MOF/COF particle size and maintenance of colloidal stability of the MOF/COF inks are crucial to prevent ink clogging. We reviewed here not only the reported MOF and COF suspensions used as inks but also the recent progress in understanding of colloidal stability of such solutions. Only recently has more insightful research based on in-depth characterization and/or modeling of colloidal MOF/COF stability started to appear. We are expecting that this understanding will grow in the coming years and can really be valorized in the formulation of MOF/COF ink formulations for direct inkjet printing.

To optimize the printing behavior of the ink, additives are regularly added to enhance the droplet formation as well as the wetting and drying behavior of the ink on the substrate. As these requirements are often specific for the application in regards to, e.g., the solvability of the MOF/COF or the droplet behavior on the substrate and the functionality of the functional materials after printing, testing of the ink during the development is highly recommended. Basic measurements of ink stability, viscosity, and surface tension as well as substrate properties like contact angle, drying, and adhesion of the ink are advised. If needed, specific additives can be used to optimize the ink for smooth droplet dynamics and high-resolution printing. Finding the right formulation is essential to balance the MOF accessibility, maintain porosity, and achieve the desired physical-mechanical stability at the desired thickness. Currently, in the field of MOFs and COFs, often only successful ink formulations are reported. To accelerate the progression of the field, we recommend that also failed ink formulations, and the related observations, be reported.

After printing, proper procedures are needed to remove solvents and potential additives from the pore space. This will involve heat and/or vacuum treatments, potentially preceded by solvent exchange (called solvent development in the context of printing) to exchange less-volatile molecules for more volatile ones. These treatments need to be carefully balanced in order to free the entire pore space but prevent framework collapse or substrate deformation and decomposition. Thereafter proper characterization of the obtained printed MOF/COF films is also needed. In addition to X-ray or electron diffraction data, a measurement of porosity is crucial to properly assess whether the crystal structure is maintained and whether the pores are still accessible after printing. As the amount of porous materials is very limited, the most commonly used techniques for assessing porosity, namely via N_2_, Ar, and CO_2_ gas adsorption isotherms, cannot be used. There are however recommended methods that can be used with thin films, namely, positron annihilation spectroscopy, krypton adsorption isotherms, ellipsometric porosity, or quartz crystal microbalance with a probe molecule.

In addition, tests should be conducted to assess the mechanical stability of the film, e.g., via scratching or tape scratching. Especially the latter is very simple to do and has been applied in several papers on inkjet printing of MOFs.^47,79,80^ We recommend that such a test be standard. While many of the works on inkjet printing of MOFs and COFs entail porous substrates, a good surface attachment may not be guaranteed, especially on smooth substrates. It is these smooth substrates (e.g., semiconductor oxides) that are especially relevant for many advanced applications in the field of sensing and electronics. Here, such tests of the mechanical stability of the film are vitally important. In that regard, we also expect that, especially for direct inkjet printing of MOFs and COFs, the use of binders (e.g., polymers) will be explored in the future to guarantee good long-term attachment. In that case, a good assessment of the accessible porosity after printing will be even more important.

Another interesting factor to consider in the future in (reactive) printing of MOFs and COFs is the possibility of postsynthetic functionalization. For instance, incorporating guest molecules or postfunctionalizing the building blocks of MOFs and COFs seems promising. Though it is not used in inkjet printing COFs yet, postsynthetic functionalization of COFs has been explored more thoroughly.^186^ Some postsynthetic functionalizations can be achieved at room temperature, which enables postsynthetic functionalization of the COF film *in situ* with a second ink for an alternative way for precise patterning of functionality in the film. Especially metal insertion and click chemistry seem very promising in this regard.

A particular strength of inkjet printing is the capability to precisely position different materials with a high spatial resolution and in a scalable manner. There are a variety of inkjet printers that can be used ranging from expensive well-controlled systems with integrated curing options to low-cost desktop inkjet printers.^49^ Typically the inkjet-printed droplets are tens of picoliters in volume. To further improve the printing resolution to femtoliter droplets, other techniques like dip pen nanolithography (DPN)^187^ where atomic force microscopy cantilever is used to dip in the ink and print by contacting the surface or microfluidic atomic force microscopy cantilever that resmbles a fountain pen could be used.^188,189^ Generally, inkjet printers have only a single nozzle and a few different cartridges. We expect that for the full potential of inkjet printing, the amount of different cartridges (and thus different inks and materials) in inkjet printing be limited to just a handful. Early work on using dual-channel nozzles, with a different precursor going through each nozzle, has shown great reduction in the probability in reactive inkjet printing of MOFs. Using multiple nozzles holds the promise of reactive printing of a large variety of multivariate MOFs/COFs.^190^ Another option will be to use a microfluidic print head with integrated mixing of different inks.^191^ Similarly, one can imagine a multichannel microfluidic mixing device integrated into the print head or ink cartridge to make direct inkjet printing of a large series of different MOFs or COFs possible.

Overall, the current work on inkjet printing of MOFs and COFs has really been able to showcase the possibility this technique offers in terms of precise position, variability of the resultant materials and gradients thereof, and diversity of substrates. For accelerated progress in the field, we recommend a better characterization and reporting of the ink formulation properties and resulting printed structures, as well as the exploration of more advanced inkjet printing hardware designs.
